# Genetic contributions to self-reported tiredness

**DOI:** 10.1038/mp.2017.5

**Published:** 2017-02-14

**Authors:** V Deary, S P Hagenaars, S E Harris, W D Hill, G Davies, D C M Liewald, International Consortium for Blood Pressure GWAS, CHARGE Consortium Aging and Longevity Group, CHARGE Consortium Inflammation Group, A M McIntosh, C R Gale, I J Deary

**Affiliations:** 1 Department of Psychology, Northumbria University, Newcastle, UK; 2 Centre for Cognitive Ageing and Cognitive Epidemiology, University of Edinburgh, Edinburgh, UK; 3 Department of Psychology, University of Edinburgh, Edinburgh, UK; 4 Division of Psychiatry, University of Edinburgh, Edinburgh, UK; 5 Medical Genetics Section, University of Edinburgh, Centre for Genomic and Experimental Medicine and MRC Institute of Genetics and Molecular Medicine, Western General Hospital, Edinburgh, UK; 6 MRC Lifecourse Epidemiology Unit, University of Southampton, Southampton, UK

## Abstract

Self-reported tiredness and low energy, often called fatigue, are associated with poorer physical and mental health. Twin studies have indicated that this has a heritability between 6 and 50%. In the UK Biobank sample (*N*=108 976), we carried out a genome-wide association study (GWAS) of responses to the question, ‘Over the last two weeks, how often have you felt tired or had little energy?’ Univariate GCTA-GREML found that the proportion of variance explained by all common single-nucleotide polymorphisms for this tiredness question was 8.4% (s.e.=0.6%). GWAS identified one genome-wide significant hit (Affymetrix id 1:64178756_C_T; *P*=1.36 × 10^−11^). Linkage disequilibrium score regression and polygenic profile score analyses were used to test for shared genetic aetiology between tiredness and up to 29 physical and mental health traits from GWAS consortia. Significant genetic correlations were identified between tiredness and body mass index (BMI), C-reactive protein, high-density lipoprotein (HDL) cholesterol, forced expiratory volume, grip strength, HbA1c, longevity, obesity, self-rated health, smoking status, triglycerides, type 2 diabetes, waist–hip ratio, attention deficit hyperactivity disorder, bipolar disorder, major depressive disorder, neuroticism, schizophrenia and verbal-numerical reasoning (absolute *r*_g_ effect sizes between 0.02 and 0.78). Significant associations were identified between tiredness phenotypic scores and polygenic profile scores for BMI, HDL cholesterol, low-density lipoprotein cholesterol, coronary artery disease, C-reactive protein, HbA1c, height, obesity, smoking status, triglycerides, type 2 diabetes, waist–hip ratio, childhood cognitive ability, neuroticism, bipolar disorder, major depressive disorder and schizophrenia (standardised *β*’s had absolute values<0.03). These results suggest that tiredness is a partly heritable, heterogeneous and complex phenomenon that is phenotypically and genetically associated with affective, cognitive, personality and physiological processes.

## Introduction

‘Hech, sirs! But I’m wabbit, I’m back frae the toon; I ha’ena dune pechin’—jist let me sit doon.’ From Glesca’ By William Dixon Cocker (1882–1970)

The present study examines genetic contributions to how the UK Biobank’s participants answered the question, ‘Over the last two weeks, how often have you felt tired or had little energy?’ Ideal questionnaire items do not have conjunctions, but the ‘or’ is understandable here, and it may even allow capture of both peripheral and central fatigue. The first and last authors of the present study grew up in South Lanarkshire in Scotland, where fatigue was often self-reported in terms of feeling ‘wabbit’. The Scots word wabbit encompasses both peripheral fatigue, the muscle weakness after a long walk, and central fatigue, the reduced ability to initiate and/or sustain mental and physical activity, such as we might experience while having flu. Throughout the paper, we refer mainly to the single English words ‘fatigue’ and/or ‘tiredness’ as the construct captured by the question, but the Scottish vernacular word is a good reminder of the subjective ‘feel’ of fatigue.

Fatigue is a common complaint. In a Dutch adult, general population survey with 9375 respondents, 4.9% reported short-term fatigue (<6 months duration), 30.5% chronic fatigue (>6 months duration) and 1% fulfilled diagnostic criteria for chronic fatigue syndrome (CFS).^1^ These findings are similar to a London-based survey of general practice patients in England, aged 18–45 years, with 15 283 respondents, where 36.7% reported substantial fatigue, 18.3% substantial fatigue of >6 months duration and 1% fulfilled the diagnostic criteria for CFS.^2^ Two other large surveys of US workers and community-dwelling adults aged 51 years and over report fatigue rates of 37.9% (2-week period prevalence) and 31.2% (1-week period prevalence) respectively.^3, 4^ In an early review of fatigue epidemiology, Lewis and Wessely^5^ argue that fatigue ‘is best viewed on a continuum’, and the continuous distribution of fatigue in the general population is supported by the Pawlikowska *et al.*^2^ study. Fatigue is also a common presentation in primary care. In a survey of 1428 consultations to 89 general practitioners in Ireland, fatigue prevalence was 25% and the main reason for attendance in 6.5%.^6^

Demographically, higher levels of self-reported fatigue are associated with female sex, lower socioeconomic status^1^ and poorer self-rated health status.^7^ There are less clear associations between age and fatigue, with some studies reporting a small but significant positive correlation between age and fatigue,^2^ whereas others report no association^6^ or a negative association.^1, 7^ There is a clearer link with the Fried phenotype of frailty in older adults,^8^ which has significant associations with mortality. The frailty phenotype comprises weakness (as measured by grip strength), weight loss, reduced mobility, reduced walking speed and fatigue.^9^

Fatigue is associated with a number of lifestyle-related factors and conditions. Smoking is a risk factor for fatigue^10^ and fatigue has strong cross-sectional associations with type 2 diabetes^11^ and increased body mass index (BMI).^12^ Fatigue is consistently associated with poorer physical and mental health status. It is one of the most common symptom complaints of cancer patients. For those undergoing treatment, prevalence estimates vary between 25 and 99%, and 25 and 30% of survivors report long-term fatigue.^13^ Fatigue is also a significant symptom of, to name just a few conditions, primary biliary cirrhosis,^14^ multiple sclerosis,^15^ rheumatoid arthritis,^16^ primary Sjogren’s syndrome^17^ and Parkinson’s disease.^18^ It is associated with chronic disease in general, and there is a linear relationship between number of chronic diseases and self-reported fatigue.^19^ Fatigue is also associated with depression,^20^ with self-reported psychological distress,^2, 21^ and with the personality trait of neuroticism.^22, 23^

Research into the biological mechanisms of fatigue has focussed on a few key areas. Fatigue is associated with the cytokine-mediated inflammatory response, particularly interleukin-1beta and interleukin-6. These latter have been shown, for instance, to be elevated in cancer patients,^24^ and administration of interferon-alpha produces depression and/or fatigue in the majority of patients receiving it as a treatment.^25^ Hypothalamic-pituitary-adrenal axis dysregulation in the form of hypocortisolaemia, blunted diurnal variation and blunted stress reactivity have been found in the cross-sectional studies of CFS patients (see Tomas *et al.*^26^ for a recent review). Other popular candidate aetio-pathological mechanisms for fatigue include serotonin pathways, circadian dysregulation, autonomic dysfunction,^17^ 5HT neurotransmitter dysregulation, alterations in ATP metabolism and vagal afferent activation.^24^ Some authors have suggested that, rather than being located with one biological system, fatigue represents a systemic dysregulation of the interaction between these systems.^27^

At the other end of the biopsychosocial spectrum, psychosocial models of fatigue focus on the role that the individual’s response to their symptom may serve in perpetuating it. For instance, in a cross-sectional study of 149 patients with multiple sclerosis, illness-related cognitions and behaviours were associated with a higher level of fatigue independent of neurological impairment.^28^ More integrative models are predicated on the notion of allostatic load, the psychophysiological work done to adjust to stress and its impact upon the body’s self-regulatory systems. As such, these models complement biological accounts of fatigue and provide potential pathways for integrating psychosocial and biological findings.^29^ Multifactorial accounts and models of fatigue exist in multiple sclerosis,^30^ primary biliary cirrhosis,^14^ obesity,^31^ diabetes,^32^ frailty^33^ and cancer.^13^ These multifactorial models postulate that fatigue is likely to be the product of physiological factors (generic, such as inflammation and/or disease specific such as hyperglycaemia in diabetes), psychosocial factors (for example, emotional distress), lifestyle and behavioural factors (for example, reduced activity), illness consequences (for example, sleep disturbance and weakness) and the interaction of these contributors.

Research into the genetics and epigenetics of fatigue has tended to focus on genes associated with the biological mechanisms described above, and done so usually within fatiguing illnesses such as those listed above. Candidate gene studies have suggested several genes to be involved in CFS, particularly genes involved in the immune system and Hypothalamic-pituitary-adrenal axis. Reviewing this literature, Landmark-Høyvik *et al.*^34^ suggest that findings are inconclusive, and are hampered by phenotypic heterogeneity, lack of power and poor study design. For example, the candidate gene studies included in the Landmark-Høyvik paper had sample sizes between 2 and 248 individuals, and the results have not been replicated. Twin studies have shown the heritability of fatigue to be between 6 and 50% with a higher concordance in monozygotic twins than dizygotic twins.^35, 36^ One of these studies^36^ did not show any sex-specific patterns of genetic influences in a Swedish sample, whereas the other one^35^ showed differences in the amount of variance explained by the genetic effect for males and females. Around half the variance in males was explained by the genetic effects compared to only a fifth of the variance in females. In a study of fatigue, insomnia and depression in 3758 twins (893 monozygotic pairs and 884 dizygotic pairs), the best model was a common pathway model, suggesting that the high association between the symptoms (correlations of 0.35–0.44) was mediated by an underlying common factor whose variation was 49% genetic and 51% unique environmental.^37^ This study showed that unique specific variance in fatigue was 38% genetic and 62% unique environmental, which supports a previous study, suggesting that fatigue is largely attributable to additive genetic factors.^38^ Genome-wide association studies (GWAS) have shown an association between single-nucleotide polymorphisms (SNPs) in genes associated with impaired cognitive abilities (*GRIK2*, *P*=1.26 × 10^−11^)^39, 40^ and the circadian clock (*NPAS2*, not genome-wide significant)^40^ and CFS, but this was in a sample of just 42 cases of CFS and 38 controls, lacking statistical power to detect genome-wide findings.

To sum up in the words of Landmark-Høyvik *et al.*:^41^ ‘fatigue can be conceptualised as a final common end point for psychological and biological processes. Fatigue is therefore both heterogeneous (occurring across different conditions) and multifactorial’. Given that, it could be argued that it is futile to search for a shared genetic contribution to tiredness, as it may not exist. However, in line with other fatigue research programmes^42^ and the research cited above, we judge that the best way to approach this complexity is to conduct large, well-designed studies focussing on specific areas of the biopsychosocial spectrum, and that to date no large study has done this at the genetic level.

Tiredness can be the result of external factors—such as poor sleep—or inherent factors—such as personality traits or poor health. It is therefore important that we clarify the scope of the present study in terms of what questions we can ask, and how definitively we can answer them. By averaging tiredness across a large sample and performing a GWAS, the present study will primarily pick upon the genetic links between tiredness and inherent factors. These are likely to be various, so we will seek to bring some clarity to what we consider to be the likely genetic heterogeneity of tiredness by posing the following questions:

1. Is there a direct genetic contribution to self-reported tiredness *per se*, not accounted for the factors in questions 2–4 below?
2. Is tiredness genetically linked to proneness to health-related traits?
3. Is tiredness genetically linked to a systemic proneness to poor health?
4. Is there a genetic relationship between the personality trait of neuroticism and tiredness?

With regard to questions 2 and 3, it is important to remember that a positive answer may constitute more than the obvious conclusion that the presence of an illness phenotype is inevitably accompanied by tiredness. Our analyses of UK Biobank data capture genetic predisposition to illness rather than its actual presence; we will address this in our polygenic prediction sensitivity analysis.

The aim of the present study, then, is to understand further the genetic contribution to self-reported tiredness and/or low energy. We conducted a genome-wide association analysis, in the UK Biobank sample, of a response to a single item question: ‘Over the past two weeks, how often have you felt tired or had little energy?’ On the basis of the foregoing literature overview, we also investigated pleiotropy with physical- and mental health-related traits, and we specifically investigated pleiotropy with factors associated with allostatic load as a first step towards answering question three above. The current study design, using >100 000 UK Biobank participants, directly addresses the main limitation from the previous studies by substantially increasing the sample size. In addition, we complement this with SNP-based heritability estimates of tiredness, sex- and age-stratified analysis, and an examination of the genetic overlap of tiredness with many health-related traits.

## Materials and methods

### Study design and participants

**Figure 1:**
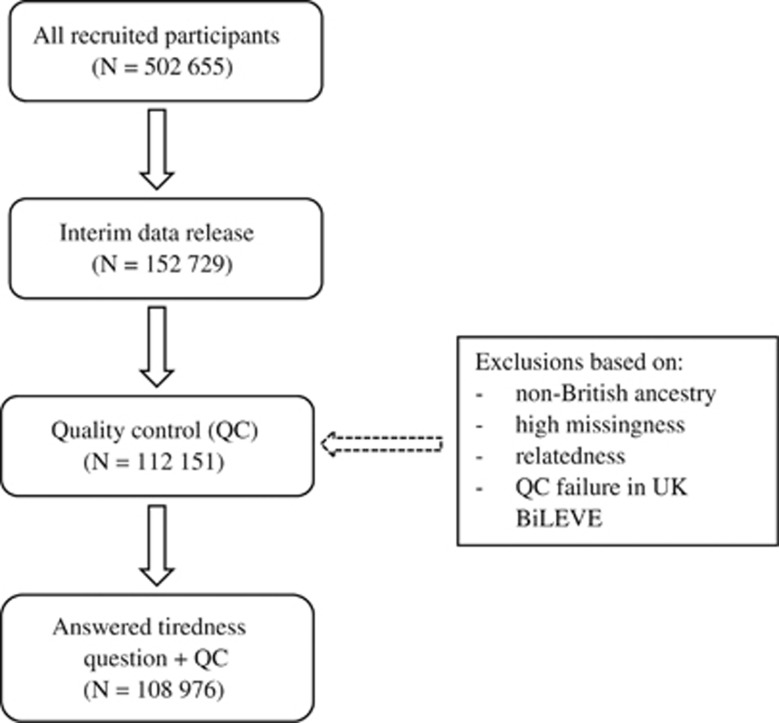
Flow diagram of participant selection.

UK Biobank is a large resource for identifying determinants of human diseases in middle-aged and older individuals (http://www.ukbiobank.ac.uk).^43^ A total of 502 655 community-dwelling individuals aged between 37 and 73 years were recruited in the United Kingdom between 2006 and 2010. Baseline assessment included cognitive testing, personality self-report, and physical and mental health measures. For the present study, genome-wide genotyping data were available for 112 151 participants (58 914 females and 53 237 males) after quality control (see below). They were aged from 40 to 73 years (mean=56.9 years, s.d.=7.9). UK Biobank received ethical approval from the Research Ethics Committee (REC reference for UK Biobank is 11/NW/0382). This study has been completed under UK Biobank application 10279. Figure 1 shows the study flow for the present report.

### Procedures

#### Tiredness

Participants were asked the question, ‘Over the past two weeks, how often have you felt tired or had little energy?’ Possible answers were: ‘Not at all/Several days/More than half the days/Nearly every day/Do not know/Prefer not to answer’. This question was asked as part of the Mental Health Questionnaire, which consists of items from the Patient Health Questionnaire.^44^ Participants answering with ‘Do not know’ or ‘Prefer not to answer’ were excluded, resulting in a four-category variable for tiredness ranging from ‘Not at all’ to ‘Nearly every day’. We will refer to this question in the rest of the paper as ‘tiredness’, but we ask the reader to bear in mind the question as it was asked, that is, its referring to tiredness and/or low energy.

#### Genotyping and quality control

The interim release of UK Biobank included genotype data for 152 729 individuals, of whom 49 979 were genotyped using the UK BiLEVE array and 102 750 using the UK Biobank axiom array. These arrays have over 95% content in common. Details of the array design, genotyping procedures and quality control details have been published elsewhere.^45, 46^

#### Imputation

An imputed data set was made available as part of the UK Biobank interim data release. The 1000 Genomes phase 3 and UK10K haplotype reference panels were merged and the genotype data imputed to this merged reference panel. Further details can be found at the following URL: http://biobank.ctsu.ox.ac.uk/crystal/refer.cgi?id=157020. Autosomal variants with a minor allele frequency ⩽0.1% and an imputation quality score of <0.1 were excluded from further analysis (*N*~17.3M SNPs).

#### Curation of summary results from GWAS consortia on health-related variables

Published summary results from international GWAS consortia were gathered to derive genetic correlations using the linkage disequilibrium (LD) score regression method and perform polygenic profile score analysis between the UK Biobank tiredness variable and the genetic predisposition to multiple health-related traits. Details of the health-related variables, the consortia’s websites, key references for each consortium and number of subjects included in each consortium’s GWAS are given in Supplementary Table 1.

### Statistical analysis

#### Phenotypic correlations

Spearman’s rank correlation coefficients were calculated between responses to the tiredness question, grip strength, forced expiratory volume in 1 s, height, BMI, self-rated health, verbal-numerical reasoning and neuroticism, all of which were measured phenotypes in UK Biobank. Details on measurements of these phenotypes can be found in the Supplementary Material.

#### Genetic association analysis

A total of 111 749 individuals answered the tiredness question and had genotypic information. After visual inspection of the distribution of the UK Biobank tiredness variable no exclusions were made. Prior to analysis, tiredness was adjusted for age, sex, assessment centre, genotyping batch and array, and 10 principal components for population stratification. Genotype–phenotype association analyses were conducted using SNPTEST v2.5.1 (ref. 47) and can be found at the following URL: https://mathgen.stats.ox.ac.uk/genetics_software/snptest/snptest.html#introduction. An additive model was specified using the ‘frequentist 1’ option. Genotype uncertainty was accounted for by analysing genotype dosages.

Genetic association analyses were also performed on the following UK Biobank phenotypes to perform further analyses: self-rated health,^48^ grip strength, forced expiratory volume in 1 s, neuroticism,^49^ verbal-numerical reasoning.^50^ We specifically examined whether any variants associated with tiredness were also associated with grip strength, self-rated health and neuroticism because we judged these to provide some coverage of physical and mental resilience in UK Biobank.

#### Estimation of SNP-based heritability

To estimate the proportion of variance explained by all common SNPs in tiredness, univariate GCTA-GREML analysis was performed.^51^ This analysis included only unrelated individuals, using a relatedness cutoff of 0.025 in the generation of the genetic relationship matrix.

#### Gene-based association analysis

Gene-based associations were derived using MAGMA,^52^ using the summary GWAS statistics for tiredness. SNPs were assigned to 18 062 genes using the National Center for Biotechnology Information build 37.3. The gene boundary was defined as the start and stop site of each gene. To account for LD between the SNPs used, the European panel of the 1000 Genomes data (phase 1, release 3) was used. A Bonferroni correction was used to control for 18 062 tests (*α*=0.05/18 062; *P*<2.768 × 10^−6^).

#### Partitioned heritability

The summary statistics from the GWAS on tiredness was partitioned into functional categories using the same data processing pipeline as Finucane *et al.*;^53^ more details on this method can be found in the Supplementary Materials.

#### Shared genetic aetiology: LD score regression and polygenic profiling

Genetic associations between tiredness and health-related variables from GWAS consortia were computed using two methods, LD score regression and polygenic profile score analysis. Each provides a different metric to infer the existence of loci contributing to pairs of traits. LD score regression was used to derive genetic correlations between two traits; this tests the degree to which the polygenic architecture of one trait overlaps with that of other traits. Polygenic profile score analysis was used to test the extent to which individual differences in the tiredness phenotype in UK Biobank could be predicted by polygenic profile scores predictive of the health-related traits from other GWAS consortia. Both of these methods are dependent each on trait being polygenic in nature, that is, where a large number of variants of small effect contribute towards phenotypic variation.^54, 55^ Bivariate LD score regression was performed between tiredness and 29 health-related traits. Polygenic profile score analysis was performed on 26 of the 29 health-related traits, as this method requires independent samples to provide the summary GWAS information from which the polygenic profile score is computed.

**Bivariate LD score regression**

This was used to quantify the extent of genetic overlap between tiredness in UK Biobank and 29 health-related traits.^55, 56^ This technique examines the correlational structure of the SNPs found across the genome. In the present study, LD score regression was used to derive genetic correlations between tiredness and health-related traits using the GWAS results of 25 large GWAS consortia and four UK Biobank phenotypes. The data processing pipeline devised by Bulik-Sullivan *et al.*^55^ was followed. To ensure that the genetic correlation for the Alzheimer’s disease phenotype was not driven by a single locus or biased the fit of the regression model, a 500-kb region centred on the *APOE* locus was removed and this phenotype was re-run. This additional model is referred to in the tables and figures as ‘Alzheimer’s disease (500 kb)’.

**Polygenic profile scores**

The UK Biobank genotyping data were recoded from numeric (1,2) allele coding to standard ACGT coding using a bespoke programme developed by one of the present authors (DCML).^46^ Polygenic profile scores were created for 25 health-related traits in all genotyped participants using PRSice.^57^ Prior to creating the scores, SNPs with a minor allele frequency <0.01 were removed and clumping was used to obtain SNPs in linkage equilibrium with an *r*^2^<0.25 within a 200 bp window. Five polygenic profile scores were created for each trait including SNPs according to their significance of association with the relevant trait at *P*-value thresholds of *P*<0.01, *P*<0.05, *P*<0.1, *P*<0.5, and all SNPs.

Regression models were used to examine the association between the polygenic profile scores and tiredness in UK Biobank, adjusting for age at measurement, sex, genotyping batch and array, assessment centre, and the first 10 principal components for population stratification. All associations were corrected for multiple testing using the false discovery rate (FDR) method.^58^ Sensitivity analyses were performed to test whether the results were confounded by individual’s neuroticism levels, their self-rated health scores or a diagnosis of major depressive disorder. This was done by adjusting the models for the neuroticism and self-rated health scores. Individuals with a probable diagnosis of major depressive disorder were excluded from the sensitivity analysis, based on the diagnostic method formulated by Smith *et al.*^59^ Further details can be found in the Supplementary Material. To examine whether any association between polygenic profile score for type 2 diabetes and tiredness was confounded by having had a diagnosis of type 2 diabetes, all individuals with a self-reported doctor’s diagnosis of type 2 diabetes were excluded from that specific sensitivity analysis (Supplementary Material). Multivariate regression was performed using all FDR significant polygenic profile scores and earlier described covariates.

#### Comparison of gene-based analysis results within UK Biobank

Gene-based associations for tiredness were compared with gene-based results for other UK Biobank health-related traits that, in the present report’s results, showed a statistically significant genetic correlation with tiredness, using previously described methods.^52^

#### Age- and sex-stratified analysis

On the basis of the age and sex distribution for tiredness (Supplementary Figure 1), further analyses examining potential age and sex effects were performed. The sample was split by sex, as well as the following three age groups for each sex: 40 to <50 years, 50 to <60 years and 60 to <70 years, one male aged >70 years was excluded from these analyses. The analysis included heritability estimates for the eight different groups, genome-wide association analysis, and genetic correlations with BMI and waist–hip ratio, as these summary data were available separately for males and females. All models were adjusted for age (sex-stratified analyses), sex (age-stratified analysis) and the previously mentioned covariates (assessment centre, genotyping batch and array, and 10 principal components for population stratification).

#### Code availability

The code used to run the analysis is available from the authors upon request.

## Results

### Phenotypic correlations

A total of 108 976 individuals from UK Biobank with genotypic data answered the question ‘Over the past two weeks, how often have you felt tired or had little energy?’, referred to hereinafter as ‘tiredness’. There were 51 416 individuals who answered ‘not at all’, 44 208 individuals responded ‘several days’, 6404 individuals answered ‘more than half the days’ and 6948 individuals responded ‘nearly every day’. Correlations indicated that individuals who reported feeling more tired tended to have lower grip strength, lower lung function, poorer self-rated health, lower scores for verbal-numerical reasoning and shorter stature (Table 1). Correlations indicated that individuals who reported feeling more tired tended to have a higher BMI and higher neuroticism scores (Table 1). Absolute effect sizes ranged from very small to moderate (Supplementary Figures 2a–f). The mean scores and distribution for each of these variables at each level of tiredness are shown in Supplementary Figure 2 and Supplementary Table 2.

### Genome-wide association study

**Figure 2:**
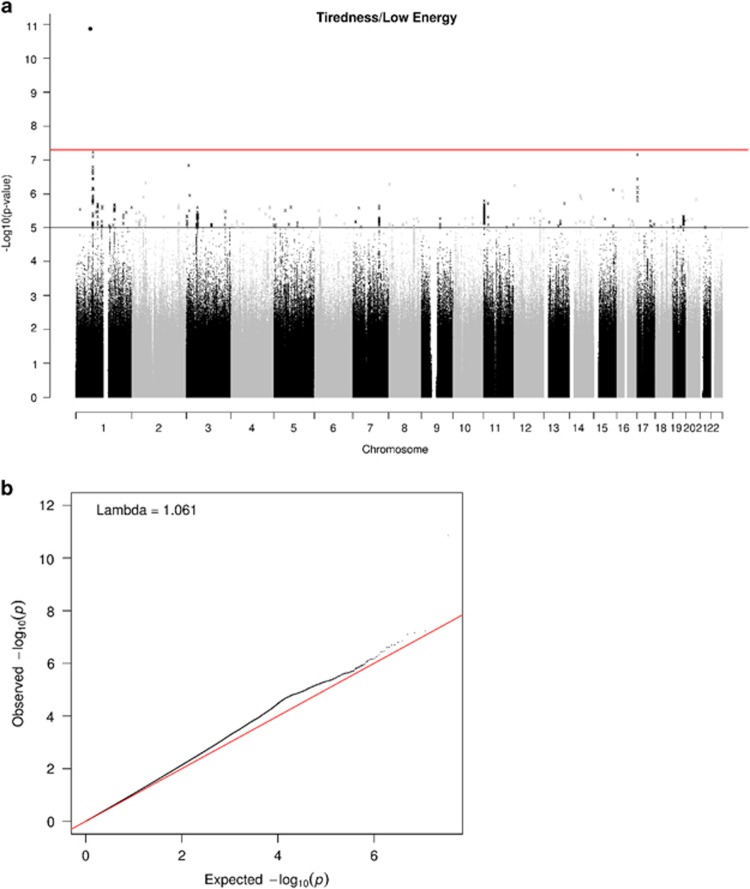
(**a**) Manhattan and (**b**) Q–Q plot of *P*-values of the SNP-based association analysis of tiredness (responses to the question, ‘Over the past two weeks, how often have you felt tired or had little energy?’). The red line on the Manhattan plot indicates the threshold for genome-wide significance (*P*<5 × 10^−8^); the grey line on the Manhattan plot indicates the threshold for suggestive significance (*P*<1 × 10^−5^). SNP, single-nucleotide polymorphism.

There was one genome-wide significant SNP (Affymetrix id 1:64178756_C_T; *P*=1.36 × 10^−11^) on chromosome 1 (Figure 2). This SNP is not in a gene and does not have an rs id. It has both a low minor allele frequency (0.001) and a low imputation quality score (0.43). It is not in a peak with other SNPs. Therefore, this result should be treated with caution. Two suggestive peaks were identified on chromosomes 1 and 17, with the lowest *P*-values of 5.88 × 10^−8^ (rs142592148; an intronic SNP in S*LC44A5*) and 6.86 × 10^−8^ (rs7219015; an intronic SNP in *PAFAH1B1*) for each peak, respectively. The peak on chromosome 1 contains three genes (*CRYZ*, *TYW3* and *SLC44A5*). The peak on chromosome 17 contains one gene (*PAFAH1B1*). The *CRY/TYW3* locus has previously been associated with circulating resistin levels, a hormone associated with insulin resistance, inflammation, and risk of type 2 diabetes and cardiovascular disease.^60^ *SLC44A5* encodes a solute carrier protein and is important for metabolism of lipids and lipoproteins, and has been associated with birth weight in cattle.^61^ *PAFAH1B1* encodes a subunit of an enzyme that has important roles in brain development and spermatogenesis. Mutations in this gene cause the neurological disorder lissencephaly.^62^

### SNP-based heritability estimate

Using GCTA-GREML common SNPs were found to explain 8.4% (s.e. 0.6%) of the phenotypic variation of tiredness as measured in UK Biobank.

### Gene-based association analysis

Gene-based association analysis identified five genes, *DRD2*, *PRRC2C*, *C3orf84*, *ANO10* and *ASXL3*, that attained genome-wide significance for tiredness (Table 2 and Supplementary Table 3) following correction for multiple comparisons. *DRD2* encodes a dopamine receptor and has previously been associated with psychiatric illnesses.^63^ Alternative splicing of *PRRC2C* has been associated with lung cancer.^64^ Mutations in *ANO10* cause cerebellar ataxias.^65^ *ASXL3* encodes a polycomb protein and mutations in this gene are associated with intellectual disability, feeding problems and distinctive facial features.^66^

In addition, each of these genes was also nominally significant in a GWAS of neuroticism, with *DRD2* being genome-wide significant in both phenotypes.^49^ A comparison with a UK Biobank GWAS of self-rated health^22^ showed that four of the five genes (*DRD2*, *PRRC2C*, *C3orf84* and *ASXL3*) were significant across phenotypes. Grip strength showed less overlap, with only *C3orf84* being nominally associated with grip strength. Of the five genes examined here, *C3orf84* was associated with each of these four phenotypes. Whereas the genetic correlations between these traits (see below) are likely to encompass multiple genic and non-genic regions, as well as unique points of overlap between pairs of phenotypes, the variants found in the *C3orf84* represent a point of the genome where the genetic architecture of these four traits converges.

### Partitioned heritability

From the full baseline model using 52 annotations, only evolutionarily conserved regions were found to be enriched for tiredness (Supplementary Figure 3). This annotation contained only 2.6% of the SNPs from the summary statistics, but they collectively explained 40% of the heritability of tiredness (s.e.=11%, enrichment metric=15.34, s.e.=4.05, *P*=0.0004). By clustering the histone marks into tissue-specific categories, we found significant enrichment for variants found in the central nervous system (Supplementary Figure 4). This category contained 15% of the SNPs and explained 45% of the heritability (s.e.=8%, enrichment metric=3.02, s.e.=0.54, *P*=0.0002).

### Genetic correlations between tiredness and physical and mental health traits

**Figure 3:**
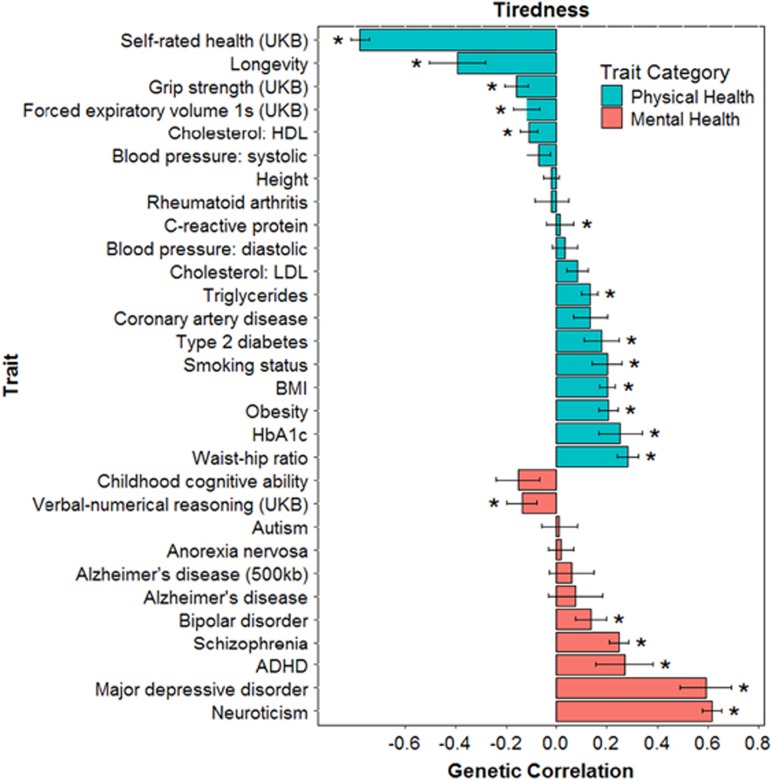
Barplot of genetic correlations (s.e.) calculated using LD regression between tiredness in UK Biobank and mental and physical health measures from GWAS consortia. *P<0.0281. ADHD, attention deficit hyperactivity disorder; BMI, body mass index; GWAS, genome-wide association study; HDL, high-density lipoprotein; LD, linkage disequilibrium; LDL, low-density lipoprotein.

LD score regression was used to test whether genetic variants associated with health-related traits also contribute towards tiredness in UK Biobank. Table 3 and Figure 3 show these genetic correlations. Positive significant (FDR corrected) genetic correlations were found between tiredness and BMI (*r*_g_=0.20), C-reactive protein (*r*_g_=0.17=0.02), HbA1c (*r*_g_=0.25), obesity (*r*_g_=0.21), smoking status (*r*_g_=0.20), triglycerides (*r*_g_=0.13), type 2 diabetes (*r*_g_=0.18), waist–hip ratio (*r*_g_=0.28), attention deficit hyperactivity disorder (*r*_g_=0.27), bipolar disorder (*r*_g_=0.14), major depressive disorder (*r*_g_=0.59), neuroticism (*r*_g_=0.62) and schizophrenia (*r*_g_=0.25). Negative significant (FDR corrected) genetic correlations were found between high-density lipoprotein (HDL) cholesterol (*r*_g_=−0.11), forced expiratory volume in 1 s (*r*_g_=−0.12), grip strength (*r*_g_=−0.16), longevity (*r*_g_=−0.39), self-rated health (*r*_g_=−0.78) and verbal-numerical reasoning (*r*_g_=−0.14). These genetic correlations suggest that there are common genetic associations between tiredness and multiple physical- and mental health-related traits. Supplementary Table 8 shows the genetic correlations between traits associated with the concept of allostatic load (blood pressure, BMI, cholesterol, C-reactive protein, HbA1c, obesity, triglycerides and waist–hip ratio), indicating genetic overlap between these traits.

### Polygenic prediction

The full results including all five thresholds can be found in Supplementary Table 4, as well as the number of SNPs included for the five thresholds in each trait. Table 4 shows the results for the polygenic profile scores analyses, using the most predictive threshold for each trait. Higher polygenic profile scores for 10 physical health traits predicted increased tiredness (significant standardised *β*’s between 0.008 and 0.026) in UK Biobank: BMI, low-density lipoprotein (LDL) cholesterol, coronary artery disease, C-reactive protein, HbA1c, obesity, smoking status, triglycerides, type 2 diabetes and waist–hip ratio. Higher polygenic profile scores for HDL cholesterol and height predicted lower tiredness (significant standardised *β*’s between *β*=−0.016 and *β*=−0.008, respectively).

Of the mental health traits, higher polygenic profile scores for bipolar disorder, neuroticism, major depressive disorder and schizophrenia were associated with increased tiredness (standardised *β*’s between 0.008 and 0.028). Polygenic profile scores for childhood cognitive ability showed a negative association with tiredness (*β*=−0.011).

Sensitivity analysis showed that, when controlling for neuroticism, the associations between tiredness and polygenic profile scores for BMI, obesity, type 2 diabetes, cholesterol (HDL and LDL), C-reactive protein, HbA1c, triglycerides, waist–hip ratio, childhood cognitive ability and schizophrenia remained significant (after FDR correction), indicating that these associations are not wholly confounded by scores for neuroticism. Similar analyses controlling for self-rated health indicated that the following associations with tiredness are not wholly confounded by self-rated health: bipolar disorder, neuroticism, major depressive disorder and schizophrenia. Supplementary Table 5 shows the adjusted results and the percentage of attenuation in standardised *β*’s for the models.

When excluding individuals with a probable diagnosis of major depressive disorder (*N*=7364) from all individuals with sufficient information about their mental health to make a probable depression diagnosis (full model, *N*=31 523), the associations between tiredness and eight polygenic profile scores remained significant (after FDR correction), compared to nine in the full model, the association between type 2 diabetes and tiredness became non-significant after excluding individuals with probable diagnosis of major depressive disorder (Supplementary Table 6). When excluding individuals with a type 2 diabetes diagnosis (*N*=725), the association between tiredness and the polygenic risk score for diabetes remained significant, indicating that this association is independent of self-reported morbidity for that disorder.

A multivariate regression model including 17 significant polygenic profile scores (BMI, HDL cholesterol, LDL cholesterol, coronary artery disease, C-reactive protein, HbA1c, height, obesity, smoking status, triglycerides, type 2 diabetes, waist–hip ratio, bipolar disorder, childhood cognitive ability, major depressive disorder, neuroticism and schizophrenia) showed that polygenic profile scores for the following traits contributed independently to the association with tiredness: BMI, HDL cholesterol, triglycerides, waist–hip ratio, childhood cognitive ability, major depressive disorder, neuroticism and schizophrenia. The scores together accounted for 0.25% of the variance in tiredness (Supplementary Table 7).

### Age- and sex-stratified analysis

**Figure 4:**
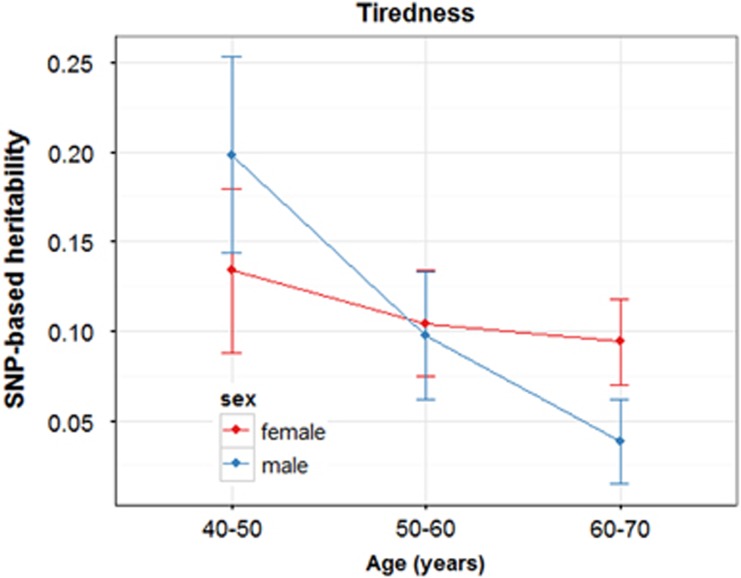
Age- and sex-stratified heritability estimates with s.e.’s for tiredness. SNP, single-nucleotide polymorphism.

GCTA-GREML analysis was used to test for possible differences in the heritability estimates for tiredness in different age/sex groups. The proportion of variance in tiredness explained by all common genetic variants using GCTA-GREML was 9.4% (s.e.=1%, *N*=57 165) in females and 8.2% (s.e.=1%, *N*=51 811) in males. Figure 4 shows heritability estimates for the three age groups in men and women (40 to <50, 50 to <60 years, and 60 to <70 years). The greatest differences can be seen between males aged 40 and 50 years (*h*^2^=19.8%, s.e.=6%, *N*=10 798) and males aged 60–70 years (*h*^2^=3.8%, s.e.=2%, *N*=24 467). Genome-wide association analysis indicated no significant sex differences between males and females, but did show some significant age differences in males (Supplementary Figures 5). Genetic correlations between BMI and tiredness were not significantly different for males (*r*_g_=0.15, s.e.=0.06, 95% confidence interval (CI)=0.04–0.26, *P*=0.0074) and females (*r*_g_=0.26, s.e.=0.05, 95% CI=0.16–0.36, *P*=4.25 × 10^−7^), as the confidence intervals were overlapping. Also, no significant differences in the genetic correlations were found between waist–hip ratio and tiredness for males (*r*_g_=0.30, s.e.=0.08, 95% CI=0.14–0.45, *P*=0.0003) and females (*r*_g_=0.271, s.e.=0.06, 95% CI=0.15–0.40, *P*=2.2 × 10^−5^), with overlapping confidence intervals.

## Discussion

In the present study, shared genetic aetiology was identified between tiredness and longevity, grip strength, multiple metabolic indicators, smoking status, neuroticism, childhood cognitive ability, depression and schizophrenia. These analyses, combining data from the UK Biobank and many GWAS consortia, provide the first estimate of the overlap in the genetic variants contributing to the heritability of tiredness and these physical and mental health-related traits and disorders. Tiredness demonstrated a significant SNP-based heritability of 8.4%.

In answer to our first research question—is there a direct genetic contribution to self-reported tiredness *per se*?—we found that, whereas there was no large influence on tiredness from common genetic variants, five genes attained genome-wide significance for tiredness: *DRD2*, *PRRC2C*, *ANO10*, *ASXL3* and *C3orf84.* The latter is an uncharacterised protein representing a point of genetic convergence between tiredness, neuroticism, grip strength and self-rated health. *DRD2*, *PRRC2C*, *ANO10* and *ASXL3* have previously been associated with psychiatric illnesses,^18^ lung cancer,^19^ cerebellar ataxias^20^ and intellectual disability,^21^ respectively. The three genes within the suggestive peak on chromosome 1 (*CRYZ*, *TYW3* and *SLC44A5*) have previously been associated with insulin resistance, inflammation, risk of type 2 diabetes, metabolism of lipids and lipoproteins, and cardiovascular disease.^15, 16^ These genes are consistent with the identification of regions associated with both tiredness and metabolic irregularities, and perhaps more broadly with ‘metabolic syndrome’ and ‘allostatic load’. *PAFAH1B*, within a suggestive peak on chromosome 17 has important roles in brain development.^17^ This is consistent with the identification of regions associated with both tiredness and cognitive traits, and the finding of significant enrichment for variants found in the central nervous system.

Evolutionarily conserved regions were found to be enriched for association with tiredness, consistent with findings for other quantitative traits including disease status,^53^ suggesting that these are important loci where common additive SNPs cluster to produce phenotypic variation in many traits, as explored in more detail in the paper by Hill *et al.*^67^ The range of factors—affective, cognitive, behavioural and physical—that are genetically associated with tiredness is in itself remarkable, and confirms the observation of Landmark-Høyvik *et al.*,^34^ quoted in the introduction, that the related construct of fatigue is aetiologically heterogeneous and multifactorial. No overlap has been found with genes (*GRIK2*, *NPAS2*) identified in previous GWAS of fatigue;^39, 40^ however, the sample size of these studies was too small to have enough power to detect statistically significant differences. The present study did not show significant associations for candidate genes previously identified.^34^

The results of the present study add to the body of evidence that tiredness has a genetic underpinning.^35, 36, 38^ This study estimated the SNP-based heritability of self-reported tiredness at 8.4%, and also examined age- and sex-specific heritability. No differences were found between males and females, but the results suggested a higher heritability in males aged between 40 and 50 years, compared to males between 60 and 70 years. Previous twin studies have shown inconsistent results regarding the sex-specific heritability. One study reported a higher heritability for prolonged fatigue (fatigue for more than one month) in males,^36^ whereas another reported a higher heritability for ‘interfering’ fatigue (fatigue for >5 days) in females.^35^ One study reported no sex differences in the heritability of chronic fatigue.^36^ In summary, the answer to our first question is that, whereas tiredness is, as expected, largely causally heterogeneous, there may be a small but significant, direct genetic contribution to tiredness proneness.

In answer to question two—is tiredness genetically linked to proneness to health-related traits?—we can answer in the affirmative. The range of factors—affective, cognitive, behavioural and physical—that are genetically associated with tiredness is in itself remarkable, and confirms the observation of Landmark-Høyvik *et al*,^34^ quoted in the introduction, that the related construct of fatigue is aetiologically heterogeneous and multifactorial. This may seem a relatively trivial finding whether we assume that it simply reflects the sum of genetic factors that are primarily associated with other more specific phenotypes, which cause tiredness in one way or another. However, it is important to recall that the biobank data capture illness propensity rather than actual morbidity. In our sensitivity analysis, we controlled for the presence of type 2 diabetes and found that the genetic link between type 2 diabetes and tiredness remained significant. This would indicate that, for this health marker at least, tiredness and illness proneness are genetically related irrespective of the presence of morbidity. Similarly, the genetic association between tiredness and longevity would argue for a non-trivial link between self-reported tiredness and a more general tendency to poor health.

This takes us into the territory of question three—is tiredness genetically linked to a systemic proneness to poor health? To begin to answer this, we examined the genetic associations between tiredness and putative markers of allostatic load. Tiredness showed significant shared heritability with a range of factors associated with the metabolic syndrome^68^ including cholesterol, triglycerides, HbA1c, waist–hip ratio, BMI, obesity and type 2 diabetes. Several of these factors are biomarkers of allostatic load.^61, 62^ The concept of allostatic load has been used in the context of both physical and mental ill health, including the symptom of fatigue. Conceptually, allostatic load represents the cumulative, physiological ‘wear and tear’ of a prolonged response to a stressor. Allostatic load has been shown to be a reliably-measurable multi-variate construct,^69, 70^ with first-order factors comprising cardiovascular, immune, metabolic, anthropometric and neuroendocrine markers.^29^ It is hypothesised that, in response to threats to homeostasis, the body’s self-regulatory mechanisms, such as the sympathetic–adrenal medullary axis and the hypothalamic pituitary adrenal axis, have the potential to ‘overcompensate and eventually collapse upon themselves’,^29^ with consequences for morbidity and mortality.

In our current analysis, we included metabolic (cholesterol, HbA1c and triglycerides), anthropometric (waist–hip ratio, BMI and obesity) and cardiovascular/respiratory (diastolic and systolic blood pressure, and forced expiratory volume) markers of allostatic load. The results showed significant shared genetic aetiology, as measured by both LD score regression and polygenic profile score analysis, between tiredness and most of the metabolic and anthropometric markers, though not the cardiovascular/respiratory markers. This raises the possibility that the genetic overlap between tiredness and these physiological factors may be due to a biological propensity to an over-compensatory physiological stress response. This suggestion will require further investigation, because a more parsimonious explanation of these links would be that there is a genetic link between tiredness and multiple, separate genetic determinants of poor physical health. However, the substantial genetic correlations between these traits, and between these traits and tiredness provide some evidence that the allostatic load concept does have coherence at the genetic level.

Fourth, to answer the question on the genetic associations between tiredness and the personality trait of neuroticism, which is the tendency to experience negative affective states, these were indeed strongly correlated, both phenotypically and genetically. This may represent a separate route to fatigue, a predominately affective one, and/or it may overlap with the physiological factors described above. A recent paper by Gale *et al.*,^49^ also using this UK Biobank sample, supports that the physiological and affective dimensions of poor health overlap in neuroticism. That paper showed that polygenic profile scores for several physical and mental health traits—BMI, coronary artery disease, smoking status, bipolar disorder, borderline personality, major depressive disorder, negative affect and schizophrenia—significantly predicted neuroticism. In the present study, when tiredness polygenic profile score analyses were adjusted for neuroticism, the associations between tiredness and mental health disorders (bar schizophrenia) were largely attenuated, whereas most of the metabolic and anthropometric associations remained significant. This suggests that it is the propensity to neuroticism, rather than the specific propensity to these disorders, that accounts or mediates the tiredness associated with mood disorders. Watson and Pennebaker,^71^ discussing competing models of how negative affectivity is related to self-reported physical and emotional well-being, found that it is associated as much with the former as with the latter, and that negative affectivity might better be conceptualised as a general tendency to experience both somatic and emotional distress. This concept of a general tendency to what they termed somatopsychic distress could explain the pleiotropy observed in the present study between neuroticism and tiredness.

That neuroticism may also be a distinct route to fatigue is supported by the fact that when the polygenic profile score analysis is adjusted for self-rated health, all associations between polygenic profile scores for physical health and tiredness are attenuated to the point of non-significance, whereas the relationship between tiredness, and polygenic profiles for neuroticism, major depressive disorder and bipolar disorder remain significant. This is consistent with the study of Gale *et al.*,^49^ investigating shared genetic aetiology between neuroticism and physical and mental health, where there were more and stronger genetic associations between neuroticism and mental health than between neuroticism and physical health. If we take self-rated health to be a marker, to some extent, of actual physical health (and the study by Harris *et al.*^48^ would indicate that it is), then this would suggest that when physical health is adjusted for, polygenic profile scores for neuroticism and its associated negative affective states, continue to make a unique contribution to tiredness.

These proposed affective and physiological routes to fatigue may not be mutually exclusive. The allostatic load model, and the multifactorial models of fatigue described in the introduction, postulate that individual differences in personality, cognition and behavioural responses to stress, and socio-cultural factors, affect the physiological stress response. An increased propensity to experience distress, as captured in the concept of neuroticism, would imply that there is increasing propensity to over-respond to stressors and thus to physiological dysregulation. In the study by Gale *et al.*,^49^ the only significant result between neuroticism and physical disease state was a significant association with the polygenic risk score for coronary artery disease, which is suggestive of an overlap of affective and cardiovascular stress responses. This multifactorial understanding of fatigue would also allow us to incorporate childhood cognitive ability (reduced ability to problem solve), smoking status (at least one study has found smoking and allostatic load interaction effects^29^) and grip strength (reduced overall system integrity/vigour) into a more general model that is suggestive of shared genetic variance between stress proneness (neuroticism, reduced cognitive ability and reduced vigour), the physiological response to stress (biomarkers), behavioural responses (smoking), self-reported tiredness, disease and mortality.^72^ However, as with our discussion of allostatic load, these links are suggestive not conclusive, and will require further empirical and theoretical investigation.

The large sample size of the present study is a strength of this study, providing powerful and robust tests of shared genetic aetiology between tiredness, and physical and mental health. A second strength is that all genetic samples were processed on the same platform at the same location. The use of summary data from many international GWAS consortia provided foundations for a comprehensive examination of shared genetic aetiology between tiredness and a wide range of health-related phenotypes.

The study has some limitations. The amount of variance explained by the polygenic profile score analysis was small, which would be expected as not all SNPs are genotyped. The SNPs that were genotyped do not necessarily accurately tag the causal genetic variants. All analyses were restricted to individuals of white British ancestry, because the sample does not have enough power to generalise results for individuals with different backgrounds. Also, the sample consisted of middle- and older-aged adults, thus limiting its generalisability to the adult population as a whole. However, as mentioned in the introduction, there are no clear age-related differences in levels of self-reported fatigue. This could be taken as an indication that the phenomenon is fairly stable across the adult life course, at least at the level of phenotype. Whether the genetic determinants are different in younger adults is a topic for future research.

A further limitation of the present study is the fact that tiredness was measured by self-report; that is, that we were looking for objective correlates of a subjective construct. However, as Wessely^73^ observed, an objective measure of fatigue is ‘an unattainable holy grail’. Almost all the studies cited in the introduction have used subjective self-reports. The self-report measures used vary widely, with there being several validated fatigue measures, and many of the reported studies use either double- or single-item questionnaires and/or single item visual analogue scales. This in itself may account for some of the inconsistency in fatigue research, though the demographic studies cited at the beginning of this article, using a wide variety of measures from single questions^7^ to a well-validated fatigue questionnaire,^2^ produced similar findings. However, our findings of genetic associations of fatigue will need replication with better validated multi-item measures.

Perhaps a more serious concern is the one signalled in the introduction: that fatigue is too causally heterogeneous a trait to meaningfully study at the genetic level. To address this, it is worth situating this research in the context of other recent and ongoing fatigue research, such as the recently announced National Institute of Health Mechanisms of Fatigue programme. The latter, while acknowledging that fatigue ‘is a common co-morbid condition in a multitude of disease conditions’, is attempting to define whether ‘molecular, cellular or imaging signatures of fatigue can be defined’.^42^ Like the psychosocial and biological fatigue research cited in our introduction, this work is predicated on the notion that, whereas fatigue shows up as a response to many physical and psychosocial stressors, its determinants may be shared across conditions. As such, we should distinguish between the effective cause of fatigue (the illness/stressors that set it going), and the material and formal causes (the bodily and psychosocial processes that produce and maintain the phenomenon).^74^ Whereas the effective causes might be various, the material and formal causes are likely to be more limited and shared across individuals and precipitating conditions. Even if this is not the case, we judge that the present study has gone some way to specifying the nature of the heterogeneity of tiredness at the genetic level, and that there are several non-trivial insights that will require further investigation, specifically: the links between tiredness and illness proneness as distinct from actual morbidity; the genetic coherence of the allostatic load concept and its contribution to tiredness; and the nature of the shared genetic and phenotypic links between tiredness and the personality trait of neuroticism. In terms of the genetic contributions to this complex phenomenon, the current study is probably best seen as the first attempt to use a large and relatively well-powered GWAS to identify these areas for future research.

### Summary

Being genetically predisposed to a range of mental and physical health complaints also predisposes individuals to report that they are more tired or lacking in energy. This study confirms that self-reported tiredness is a partly heritable, heterogeneous and complex phenomenon that is phenotypically and genetically associated with affective, cognitive, personality, health and physiological processes. This study also served as a first step in testing some genetic hypotheses from the allostatic load model, finding suggestive links between tiredness and three genes on chromosome one associated with allostatic processes and considerable genetic overlap between tiredness and allostatic markers. We can foresee more tests of these links as more genome-wide genotyping data become available.

## Figures and Tables

**Table 1:** Spearman phenotypic correlations between tiredness (responses to the question, ‘Over the past two weeks, how often have you felt tired or had little energy?’) and physical and mental health

	*Tiredness*
Self-rated health (*N*=108 648)	−0.35
Grip strength (*N*=108 573)	−0.12
Forced expiratory function in 1 s (*N*=101 823)	−0.08
Height (*N*=108 796)	−0.09
BMI (*N*=108 681)	0.10
Verbal-numerical reasoning (*N*=35 101)	−0.04
Neuroticism (*N*=105 456)	0.39

Abbreviation: BMI, body mass index.

All correlations had *P*<0.001.

**Table 2:** The genome-wide significant genes from the UK Biobank tiredness phenotype and the significance values for the same genes using the neuroticism, SRH and grip phenotypes, also in the UK Biobank sample

*CHR*	*Gene*	*Tiredness* P	*SRH* P	*Grip* P	*Neuroticism* P
11	*DRD2*	2.94 × 10^−7^	0.012	0.156	9.69 × 10^−9^
1	*PRRC2C*	1.43 × 10^−6^	0.002	0.314	0.020
3	*C3orf84*	1.45 × 10^−6^	7.38 × 10^−5^	0.001	0.016
3	*ANO10*	1.52 × 10^−6^	0.058	0.728	0.001
18	*ASXL3*	2.67 × 10^−6^	1.03^−6^	0.052	1.36 × 10^−5^

Abbreviations: grip, grip strength; SRH, self-rated health.

Unmodified *P*-values are shown for all phenotypes.

**Table 3:** Genetic correlations between tiredness documented in the UK Biobank data set and the health-related variables collected from GWAS consortia

*Trait category*	*Traits from GWAS consortia*	r_*g*_	*s.e.*	P
Physical health	Blood pressure: diastolic	0.0332	0.0502	0.5083
	Blood pressure: systolic	−0.0698	0.0478	0.1444
	BMI	0.2024	0.0322	**3.18 × 10**^**−10**^
	Cholesterol: HDL	−0.1087	0.0373	**0.0036**
	Cholesterol: LDL	0.0829	0.0413	0.0449
	Coronary artery disease	0.1338	0.067	0.0459
	C-reactive protein	0.0165	0.054	**0.0021**
	Grip strength^a^	−0.1596	0.0482	**0.0009**
	HbA1c	0.2536	0.0857	**0.0031**
	Height	−0.0201	0.0297	0.4980
	Longevity	−0.3943	0.1096	**0.0003**
	Forced expiratory volume 1s^a^	−0.1181	0.0538	**0.0281**
	Obesity	0.2063	0.0381	**6.31 × 10**^**−8**^
	Rheumatoid arthritis	−0.0181	0.0674	0.7885
	Self-rated health^a^	−0.7780	0.0349	**7.30 × 10**^**−110**^
	Smoking status	0.2009	0.0603	**0.0009**
	Triglycerides	0.1324	0.0332	**6.62 × 10**^**−5**^
	Type 2 diabetes	0.1784	0.0689	**0.0097**
	Waist–hip ratio	0.2834	0.0417	**1.09 × 10**^**−11**^
Mental health	ADHD	0.2694	0.1116	**0.0158**
	Alzheimer’s disease	0.0762	0.1079	0.4801
	Alzheimer’s disease (500 kb)	0.0613	0.0872	0.4816
	Anorexia nervosa	0.0192	0.0492	0.6967
	Autism	0.0129	0.0695	0.8522
	Bipolar disorder	0.1382	0.0605	**0.0223**
	Childhood cognitive ability	−0.1528	0.0891	0.0864
	Major depressive disorder	0.5902	0.1015	**6.03 × 10**^**−9**^
	Neuroticism	0.6150	0.038	**7.34 × 10**^**−59**^
	Schizophrenia	0.2490	0.0386	**1.14 × 10**^**−10**^
	Verbal-numerical reasoning^a^	−0.1379	0.0596	**0.0206**

Abbreviations: ADHD, attention deficit hyperactivity disorder; BMI, body mass index; FDR, false discovery rate; GWAS, genome-wide association study; HDL, high-density lipoprotein; LDL, low-density lipoprotein.

Statistically significant *P*-values (after false discovery rate correction; threshold: *P*=0.0281) are shown in bold.

a GWAS based on UK Biobank data.

**Table 4:** Associations between polygenic profile scores of health-related traits created from GWAS consortia summary data, and the UK Biobank tiredness phenotype controlling for age, sex, assessment centre, genotyping batch, and array and 10 principal components for population structure

*Trait category*	*Trait*	*Threshold*	β	P
Physical health	Blood pressure: diastolic	0.1	−0.0028	0.3619
	Blood pressure: systolic	0.1	−0.0025	0.4077
	BMI	1	0.0280	**4.90 × 10**^−**20**^^a^
	Cholesterol: HDL	0.5	−0.0163	**8.49 × 10**^−**8**^^a^
	Cholesterol: LDL	0.5	0.0081	**0.0077**^a^
	Coronary artery disease	0.5	0.0084	**0.0061**
	C-reactive protein	1	0.0130	**2.10 × 10**^−**5**^^a^
	Forced expiratory volume 1 s	0.01	−0.0059	0.0529
	Longevity	0.05	−0.0067	0.0297
	HbA1c	1	0.0090	**0.0033**^a^
	Height	1	−0.0077	**0.0154**
	Obesity	1	0.0236	**1.20 × 10**^−**14**^^a^
	Rheumatoid arthritis	0.1	−0.0016	0.5926
	Smoking status	0.5	0.0086	**0.0071**
	Triglycerides	0.5	0.0209	**1.06 × 10**^−**11**^^a^
	Type 2 diabetes	1	0.0120	**0.0002**^a^,^b^
	Waist–hip ratio	1	0.0258	**7.85 × 10**^−**17**^^a^
Mental health	ADHD	1	0.0042	0.1647
	Alzheimer’s disease	0.05	−0.0052	0.0889
	Anorexia nervosa	0.5	0.0048	0.1169
	Autism	1	−0.0018	0.5593
	Bipolar disorder	0.01	0.0081	**0.0076**^c^
	Childhood cognitive ability	0.1	−0.0112	**0.0002**^a^
	Major depressive disorder	1	0.0185	**2.25 × 10**^−**9**^^c^
	Neuroticism	0.1	0.0183	**2.00 × 10**^−**9**^^c^
	Schizophrenia	1	0.0283	**2.31 × 10**^−**19**^^a^,^c^

Abbreviations: ADHD, attention deficit hyperactivity disorder; BMI, body mass index; FDR, false discovery rate; GWAS, genome-wide association study; HDL, high-density lipoprotein; LDL, low-density lipoprotein.

FDR-corrected statistically significant values (*P*=0.0255) are shown in bold. The associations between the polygenic profile scores with the largest effect size (threshold) and tiredness are presented. Threshold is the *P*-value threshold with the largest effect size.

a Results remain significant after controlling for neuroticism scores.

b Results remain significant after excluding individuals with type 2 diabetes (*β*=0.0105, *P*=0.00076).

c Results remain significant after controlling for self-rated health.
