# Erratum: Genetic contributions to self-reported tiredness

**DOI:** 10.1038/mp.2017.70

**Published:** 2017-03-21

**Authors:** V Deary, S P Hagenaars, S E Harris, W D Hill, G Davies, D C M Liewald, International Consortium for Blood Pressure GWAS, CHARGE consortium Aging and Longevity Group, CHARGE consortium Inflammation Group, A M McIntosh, C R Gale, I J Deary

**Keywords:** Genome-wide association studies, Fatigue, Clinical genetics

**Correction to:** *Molecular Psychiatry* (2017) advance online publication 14 February 2017; doi:10.1038/mp.2017.5

Following publication of this paper, the authors noticed a mistake in Tables 3 and 4 and Figure 3. The genetic correlation between tiredness and C-reactive protein was displayed as 0.0165, but the correct genetic correlation is 0.1650. The thresholds for major depressive disorder and neuroticism are incorrect in Table 4. The threshold for major depressive disorder is 0.1, and for neuroticism the threshold is 1. The corrected tables and figure appear below.

**Table 3:** Genetic correlations between tiredness documented in the UK Biobank data set and the health-related variables collected from GWAS consortia

*Trait category*	*Traits from GWAS consortia*	*rg*	*s.e.*	P*-value*
Physical health	Blood pressure: diastolic	0.0332	0.0502	0.5083
	Blood pressure: systolic	−0.0698	0.0478	0.1444
	BMI	0.2024	0.0322	**3.18 × 10** ^**−10**^
	Cholesterol: HDL	−0.1087	0.0373	**0.0036**
	Cholesterol: LDL	0.0829	0.0413	0.0449
	Coronary artery disease	0.1338	0.067	0.0459
	C-reactive protein	0.1650	0.054	**0.0021**
	Grip strength^a^	−0.1596	0.0482	**0.0009**
	HbA1c	0.2536	0.0857	**0.0031**
	Height	−0.0201	0.0297	0.4980
	Longevity	−0.3943	0.1096	**0.0003**
	Forced expiratory volume 1s^a^	−0.1181	0.0538	0.0281
	Obesity	0.2063	0.0381	**6.31 × 10** ^**−8**^
	Rheumatoid arthritis	−0.0181	0.0674	0.7885
	Self-rated health^a^	−0.7780	0.0349	**7.30 × 10** ^**−110**^
	Smoking status	0.2009	0.0603	**0.0009**
	Triglycerides	0.1324	0.0332	**6.62 × 10** ^**−5**^
	Type 2 diabetes	0.1784	0.0689	**0.0097**
	Waist–hip ratio	0.2834	0.0417	**1.09 × 10** ^**−11**^
Mental health	ADHD	0.2694	0.1116	**0.0158**
	Alzheimer's disease	0.0762	0.1079	0.4801
	Alzheimer’s disease (500 kb)	0.0613	0.0872	0.4816
	Anorexia nervosa	0.0192	0.0492	0.6967
	Autism	0.0129	0.0695	0.8522
	Bipolar disorder	0.1382	0.0605	**0.0223**
	Childhood cognitive ability	−0.1528	0.0891	0.0864
	Major depressive disorder	0.5902	0.1015	**6.03 × 10** ^**−9**^
	Neuroticism	0.6150	0.038	**7.34 × 10** ^**−59**^
	Schizophrenia	0.2490	0.0386	**1.14 × 10** ^**−10**^
	Verbal–numerical reasoning^a^	−0.1379	0.0596	**0.0206**

Abbreviations: ADHD, attention deficit hyperactive disorder; BMI, body mass index; GWAS, genome-wide association study; HbA1c, hemoglobin A1c; HDL, high-density lipoproteins; LDL, low-density lipoproteins; rg, genetic correlation; s.e., standard error.

Statistically significant *P*-values (after false discovery rate correction; threshold: *P*=0.0281) are shown in bold.

^a^GWAS based on UK Biobank data.

**Table 4:** Associations between polygenic profile scores of health-related traits created from GWAS consortia summary data, and the UK Biobank tiredness phenotype controlling for age, sex, assessment centre, genotyping batch and array, and 10 principal components for population structure

*Trait category*	*Trait*	*Threshold*	β	P*-value*
Physical health	Blood pressure: diastolic	0.1	−0.0028	0.3619
	Blood pressure: systolic	0.1	−0.0025	0.4077
	BMI	1	0.0280	**4.90** × **10**^−**20**^^a^
	Cholesterol: HDL	0.5	−0.0163	**8.49** × **10**^−**8**^^a^
	Cholesterol: LDL	0.5	0.0081	**0.0077** ^a^
	Coronary artery disease	0.5	0.0084	**0.0061**
	C-reactive protein	1	0.0130	**2.10** × **10**^−**5**^^a^
	Forced expiratory volume 1s	0.01	−0.0059	0.0529
	Longevity	0.05	−0.0067	0.0297
	HbA1c	1	0.0090	**0.0033** ^a^
	Height	1	−0.0077	**0.0154**
	Obesity	1	0.0236	**1.20** × **10**^−**14**^^a^
	Rheumatoid arthritis	0.1	−0.0016	0.5926
	Smoking status	0.5	0.0086	**0.0071**
	Triglycerides	0.5	0.0209	**1.06** × **10**^−**11**^^a^
	Type 2 diabetes	1	0.0120	**0.0002**^a^,^b^
	Waist–hip ratio	1	0.0258	**7.85** × **10**^−**17**^^a^
Mental health	ADHD	1	0.0042	0.1647
	Alzheimer’s disease	0.05	−0.0052	0.0889
	Anorexia nervosa	0.5	0.0048	0.1169
	Autism	1	−0.0018	0.5593
	Bipolar disorder	0.01	0.0081	**0.0076** ^c^
	Childhood cognitive ability	0.1	−0.0112	**0.0002** ^a^
	Major depressive disorder	0.1	0.0185	**2.25** × **10**^−**9**^^c^
	Neuroticism	1	0.0183	**2.00** × **10**^−**9**^^c^
	Schizophrenia	1	0.0283	**2.31** × **10**^−**19**^^a^,^c^

Abbreviations: ADHD, attention deficit hyperactive disorder; BMI, body mass index; GWAS, genome-wide association study; HbA1c, hemoglobin A1c; HDL, high-density lipoproteins; LDL, low-density lipoproteins.

False discovery rate-corrected statistically significant values (*P*=0.0255) are shown in bold. The associations between the polygenic profile scores with the largest effect size (threshold) and tiredness are presented. Threshold is the *P*-value threshold with the largest effect size.

^a^Results remain significant after controlling for neuroticism scores.

^b^Results remain significant after excluding individuals with type 2 diabetes (*β*=0.0105, *P*=0.00076).

^c^Results remain significant after controlling for self-rated health.

**Figure 1:**
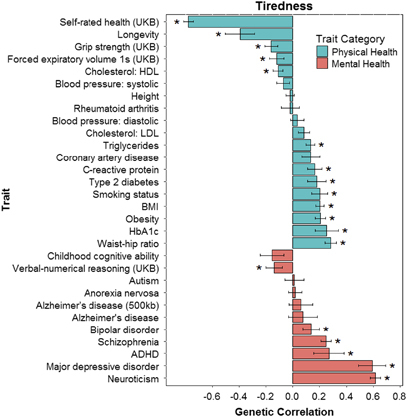
Barplot of genetic correlations (s.e.) calculated using linkage disequilibrium score regression between tiredness in UK Biobank, and mental and physical health measures from GWAS consortia. *P< 0.0281. ADHD, attention deficit hyperactive disorder; BMI, body mass index; GWAS, genome-wide association study; HbA1c, hemoglobin A1c; HDL, high-density lipoproteins; LDL, low-density lipoproteins. PowerPoint slide

## PowerPoint slides

PowerPoint slide for Fig. 1

