# Supplementary material for: Genetic contributions to self-reported tiredness
Source: Mol Psychiatry. 2017 Feb 14;23(3):609–20. doi: 10.1038/mp.2017.5 (PMC5822465; doi:10.1038/mp.2017.5)
Supplement: Supplementary Material [file mp20175x1.docx]

**Supplementary Materials for:**

Genetic contributions to self-reported tiredness/energy.

Contents

1. Supplementary Methods

Phenotypic measures

Sources of genetic results from genome-wide association consortia

Stratified linkage disequilibrium score regression

Sensitivity analysis

1. Supplementary Tables and Figures

Supplementary Table 1: Details of the sources of genetic results from genome-wide association studies (GWAS) consortia.

Supplementary Figure 1. Age- and sex distribution of standardized tiredness scores

Supplementary Table 2: Frequency table for tiredness and self-rated health

Supplementary Figure 2a-f: Distributions of different phenotypic measures at each level of tiredness.

Supplementary Table 3: Genome-wide significant gene-based hits

Supplementary Figure 3: Enrichment analysis for tiredness using functional categories

Supplementary Figure 4: Enrichment analysis for tiredness using cell specific functional categories.

Supplementary Table 4: Complete polygenic profile score associations with tiredness using all five thresholds.

Supplementary Table 5. Polygenic profile score associations with tiredness using all five thresholds adjusted for self-rated health and neuroticism.

Supplementary Table 6. Polygenic profile score associations with tiredness using all five thresholds adjusted for major depressive disorder.

Supplementary Table 7. Multivariate model with all significant polygenic profile scores predicting tiredness.

Supplementary Table 8. Genetic correlations between allostatic load traits

Supplementary Figure 5: Miami and QQ plot for tiredness split by sex

Supplementary Figure 6: Manhattan plot of the difference in effect size between males and females.

Supplementary Figure 7: Miami and QQ plot for tiredness split by men aged 40-50 years and men aged 60-70 years.

Supplementary Figure 8: Manhattan plot of the difference in effect size between by men aged 40-50 years and men aged 60-70 years.

**Phenotypic measures**

**Grip strength**

Right and left hand grip strength were measures using a Jamar J00105 hydraulic hand dynamometer. A dominant grip strength measure was computed based on handedness.

**Forced expiratory volume in 1s**

Forced expiratory volume in one second was measured using a Vitalograph Pneumotrac 6800. Each participant was asked to record two to five blows, lasting for a minimum of six seconds, within a period of six minutes. The reproducibility of the first two blows was compared and if there was less than 5% difference in forced volume vital capacity and forced expiratory volume in one second, a third blow was not required.

**Height**

Standing height was measured in cm using a Seca 202 device.

**BMI**

BMI values in kg/m^2^ were constructed from height and weight measures.

**Self-rated health**

Participants were asked the question, “In general how would you rate your overall health?”. Possible answers were “Excellent/Good/Fair/Poor/Do not know/Prefer not to answer”. We created a four-category SRH variable indexing how each participant rated their health ranging from “excellent” to “poor”; excluding those that responded with “do not know” or “prefer not to answer”. A higher score for SRH indicates a better health rating (Harris et al., 2015).

**Verbal-numerical reasoning**

Verbal-numerical reasoning was measured by a thirteen item-test with a time limit of two minutes, completed by 36 035 individuals. Six items were verbal and seven numerical. An example of a verbal question is ‘Bud is to flower as child is to?’ (Possible answers: ‘Grow/Develop/Improve/Adult/Old/Do not know/Prefer not to answer’). An example of a numerical question is ‘If sixty is more than half of seventy-five, multiply twenty-three by three. If not subtract 15 from eighty-five. Is the answer?’ (Possible answers: ‘68/69/70/71/72/Do not know/Prefer not to answer’). The verbal-numerical reasoning score was the total score out of thirteen (Hagenaars et al., 2016).

**Neuroticism**

Participants completed 12 questions of the Eysenck Personality Questionnaire-Revised Short Form (EPQ-R Short Form) neuroticism scale (Deary & Bedford, 2011; Eysenck, Eysenck, & Barrett, 1985). Neuroticism refers to the relatively stable personality trait that assesses individual differences in the tendency to experience negative emotions. A summary score was derived to obtain a measure of neuroticism (Gale et al., In Press).

**Sources of genetic results from genome-wide association consortia**

**CARDIoGRAM**

Coronary artery disease data have been contributed by CARDIoGRAMplusC4D investigators.

**CHARGE-Aging and Longevity**

Longevity data have been provided by the CHARGE-Aging and Longevity consortium. Longevity was defined as reaching age 90 years or older. Genotyped participants who died between the ages of 55 and 80 years were used as the control group. There were 6036 participants who achieved longevity and 3757 participants in the control group across participating studies in the discovery meta-analysis.

Broer L, Buchman AS, Deelen J, Evans DS, Faul JD, Lunetta KL, Sebastiani P, Smith JA, Smith AV, Tanaka T, Yu L, Arnold AM, Aspelund T, Benjamin EJ, De Jager PL, Eirkisdottir G, Evans DA, Garcia ME, Hofman A, Kaplan RC, Kardia SL, Kiel DP, Oostra BA, Orwoll ES, Parimi N, Psaty BM, Rivadeneira F, Rotter JI, Seshadri S, Singleton A, Tiemeier H, Uitterlinden AG, Zhao W, Bandinelli S, Bennett DA, Ferrucci L, Gudnason V, Harris TB, Karasik D, Launer LJ, Perls TT, Slagboom PE, Tranah GJ, Weir DR, Newman AB, van Duijn CM and Murabito JM. **GWAS of Longevity in CHARGE Consortium Confirms APOE and FOXO3 Candidacy**. *J Gerontol A Biol Sci Med Sci*. 2015;70:110-8.

*Acknowledgments*

The CHARGE Aging and Longevity working group analysis of the longevity phenotype was funded through the individual contributing studies. The working group thanks all study participants and study staff**.**

**CHARGE-Inflammation Working Group**

C-reactive protein data have been provided by the CHARGE-Inflammation Working Group.

Abbas Dehghan, Josée Dupuis, Maja Barbalic, Joshua C. Bis, Gudny Eiriksdottir, Chen Lu, Niina Pellikka, Henri Wallaschofski, Johannes Kettunen, Peter Henneman, Jens Baumert, David P. Strachan, Christian Fuchsberger, Veronique Vitart, James F. Wilson, Guillaume Paré, Silvia Naitza, Megan E. Rudock, Ida Surakka, Eco J.C. de Geus, Behrooz Z. Alizadeh, Jack Guralnik, Alan Shuldiner, Toshiko Tanaka, Robert Y.L. Zee, Renate B. Schnabel, Vijay Nambi, Maryam Kavousi, Samuli Ripatti, Matthias Nauck, Nicholas L. Smith, Albert V. Smith, Jouko Sundvall, Paul Scheet, Yongmei Liu, Aimo Ruokonen, Lynda M. Rose, Martin G. Larson, Ron C. Hoogeveen, Nelson B. Freimer, Alexander Teumer, Russell P. Tracy, Lenore J. Launer, Julie E. Buring, Jennifer F. Yamamoto, Aaron R. Folsom, Eric J.G. Sijbrands, James Pankow, Paul Elliott, John F. Keaney, Wei Sun, Antti-Pekka Sarin, João D. Fontes, Sunita Badola, Brad C. Astor, Albert Hofman, Anneli Pouta, Karl Werdan, Karin H. Greiser, Oliver Kuss, Henriette E. Meyer zu Schwabedissen, Joachim Thiery, Yalda Jamshidi, Ilja M. Nolte, Nicole Soranzo, Timothy D. Spector, Henry Völzke, Alexander N. Parker, Thor Aspelund, David Bates, Lauren Young, Kim Tsui, David S. Siscovick, Xiuqing Guo, Jerome I. Rotter, Manuela Uda, David Schlessinger, Igor Rudan, Andrew A. Hicks, Brenda W. Penninx, Barbara Thorand, Christian Gieger, Joe Coresh, Gonneke Willemsen, Tamara B. Harris, Andre G. Uitterlinden, Marjo-Riitta Järvelin, Kenneth Rice, Dörte Radke, Veikko Salomaa, Ko Willems van Dijk, Eric Boerwinkle, Ramachandran S. Vasan, Luigi Ferrucci, Quince D. Gibson, Stefania Bandinelli, Harold Snieder, Dorret I. Boomsma, Xiangjun Xiao, Harry Campbell, Caroline Hayward, Peter P. Pramstaller, Cornelia M. van Duijn, Leena Peltonen, Bruce M. Psaty, Vilmundur Gudnason, Paul M. Ridker, Georg Homuth, Wolfgang Koenig, Christie M. Ballantyne, Jacqueline C.M. Witteman, Emelia J. Benjamin, Markus Perola and Daniel I. Chasman. **Meta-Analysis of Genome-Wide Association Studies in >80 000 Subjects Identifies Multiple Loci for C-Reactive Protein Levels.** *Circulation* 2011: 123: 731-738.

Acknowledgments

The CHARGE Inflammation Working Group analysis of C-reactive protein was funded through the individual contributing studies. The working group thanks all study participants and study staff.

**CHIC**

Childhood cognitive ability data were obtained from the CHIC consortium.

**DIAGRAM**

Type 2 diabetes data were obtained from the DIAGRAM consortium.

**Genetic Consortium for Anorexia nervosa**

Anorexia nervosa data were obtained from the Genetic Consortium for Anorexia nervosa.

**Global Lipids Consortium**

LDL cholesterol, HDL cholesterol and triglycerides data have been contributed by the Global Lipids Consortium.

**GIANT**

BMI, height, waist-hip ratio and obesity data were obtained from the GIANT consortium.

**International Consortium for Blood Pressure (ICBP)**

Blood pressure data were provided by ICBP.

**International Genomics of Alzheimer’s Project (IGAP)**

Alzheimer’s disease data were obtained from (IGAP)

*Material and methods*

International Genomics of Alzheimer's Project (IGAP) is a large two-stage study based upon genome-wide association studies (GWAS) on individuals of European ancestry. In stage 1, IGAP used genotyped and imputed data on 7 055 881 single nucleotide polymorphisms (SNPs) to meta-analyse four previously-published GWAS datasets consisting of 17 008 Alzheimer's disease cases and 37 154 controls (The European Alzheimer's disease Initiative – EADI the Alzheimer Disease Genetics Consortium – ADGC The Cohorts for Heart and Aging Research in Genomic Epidemiology consortium – CHARGE The Genetic and Environmental Risk in AD consortium – GERAD). In stage 2, 11 632 SNPs were genotyped and tested for association in an independent set of 8572 Alzheimer's disease cases and 11 312 controls. Finally, a meta-analysis was performed combining results from stages 1 & 2.

*Acknowledgments*

We thank the International Genomics of Alzheimer's Project (IGAP) for providing summary results data for these analyses. The investigators within IGAP contributed to the design and implementation of IGAP and/or provided data but did not participate in analysis or writing of this report. IGAP was made possible by the generous participation of the control subjects, the patients, and their families. The i–Select chips was funded by the French National Foundation on Alzheimer's disease and related disorders. EADI was supported by the LABEX (laboratory of excellence program investment for the future) DISTALZ grant, Inserm, Institut Pasteur de Lille, Université de Lille 2 and the Lille University Hospital. GERAD was supported by the Medical Research Council (Grant n° 503480), Alzheimer's Research UK (Grant n° 503176), the Wellcome Trust (Grant n° 082604/2/07/Z) and German Federal Ministry of Education and Research (BMBF): Competence Network Dementia (CND) grant n° 01GI0102, 01GI0711, 01GI0420. CHARGE was partly supported by the NIH/NIA grant R01 AG033193 and the NIA AG081220 and AGES contract N01–AG–12100, the NHLBI grant R01 HL105756, the Icelandic Heart Association, and the Erasmus Medical Center and Erasmus University. ADGC was supported by the NIH/NIA grants: U01 AG032984, U24 AG021886, U01 AG016976, and the Alzheimer's Association grant ADGC–10–196728.

**MAGIC**

Data on glycaemic traits have been contributed by MAGIC investigators.

**Psychiatric Genetics Consortium**

Schizophrenia, bipolar disorder, major depressive disorder and ADHD and autism data were obtained from the Psychiatric Genetics Consortium.

**Rheumatoid Arthritis**

Rheumatoid arthritis data were obtained from the Broad Institute

**SpiroMeta/CHARGE Pulmonary**

Lung function data were obtained from the SpiroMeta and CHARGE Pulmonary group.

**Tobacco and Genetic Consortium**

Smoking status data were obtained from the Tobacco and Genetics Consortium.

**The Genetics of Personality Consortium**

Neuroticism data were obtained from the Genetics of Personality consortium.

**Stratified linkage disequilibrium score regression**

The summary statistics from the GWAS on tiredness/little energy were portioned into functional categories using the same data processing pipeline as Finucane et al., (Finucane et al., 2015).

The heritability Z-score for the tiredness/little energy phenotype was 9.84 indicating a sufficient level of polygenic signal for use with stratified LD score regression. We first derive the heritability for each of the functional annotations whilst controlling for LD and for the level of overlap between the functional annotations used. Using the heritability of each of the functional annotations we then derive an enrichment metric defined as Pr(h^2)/Pr(SNPs), or the proportion of the heritability, over the proportion of SNPs found in the annotation. This describes the degree to which the annotation is enriched for variants that are associated with tiredness/little energy. LD scores were calculated using the European samples from the 1000 Genomes project (1000G) and only included the HapMap 3 SNPs with a minor allele frequency (MAF) of >0.05. For controlling for multiple testing a False discovery rate (FDR) (Benjamini & Hochberg, 1995) was applied to the full baseline model (52 tests). For the tissue specific analysis, 10 tests were controlled for using FDR correction.

**Sensitivity analyses**

Sensitivity analyses were performed by excluding individuals with a diagnosis for type 2 diabetes (n = 725) from the regression models between tiredness and polygenic risk for type 2 diabetes. The diagnosis for type 2 diabetes was based on a verbal interview with a nurse, which followed after a touchscreen questionnaire asking about history of illnesses. If a participant had answered in the touchscreen questionnaire that they had diabetes (any type) the nurse would confirm this and amend if incorrectly answered in the touchscreen questionnaire.

Sensitivity analysis for major depressive disorder were performed in a different way. When excluding individuals with a probable diagnosis of major depressive disorder, based on the criteria by (Smith et al., 2013), individuals with missing data on the mood questions are not considered, even though they might have diagnosis of major depressive disorder. Therefore, all polygenic profile analysis were rerun in individuals with sufficient information to make a probable diagnosis of major depressive disorder (N = 31 523), followed by excluding individuals with a probable diagnosis of major depressive disorder (N = 7364) from this full set.

**Supplementary Table 1**

Sources of genetic results from genome-wide association consortia

| **Phenotype** | **Consortium** | **URL** | **Reference** | **No. of individuals in GWAS** |
| --- | --- | --- | --- | --- |
| Childhood cognitive ability | CHIC | http://ssgac.org/Data.php | Benyamin et al. Mol Psychiatr 2014; 19: 253-258. PMID: 23358156 | 17 989 |
| Neuroticism | The Genetics of Personality Consortium | http://www.tweelingenregister.org/GPC/ | De Moor, et al. JAMA Psychiatry 2015; 72(7):642-650. PMID: 25993607 | 63 661 |
| BMI | GIANT | http://www.broadinstitute.org/collaboration/ giant/index.php/GIANT_consortium_data_files | Locke et al. Nature 2015; 518: 197-206. PMID: 25673413 | 339 224 |
| Height | GIANT | http://www.broadinstitute.org/collaboration/giant/index.php/GIANT_consortium_data_files | Wood et al. Nature Genetics 2014; 11:1173-86 | 253 288 |
| Obesity | GIANT | http://www.broadinstitute.org/collaboration/giant/index.php/GIANT_consortium_data_files | Berndt et al. Nature Genetics 2013; 45:501-512 | 263 407 |
| Waist-hip ratio | GIANT | http://www.broadinstitute.org/collaboration/giant/index.php/GIANT_consortium_data_files | Shungin et al. Nature 2015; 518: 187-196 | 224 459 |
| Longevity | CHARGE-Aging and Longevity working group |  | Broer et al. J Gerontol A Biol Sci Med Sci 2015; 70: 110-118. PMID: 25199915 | 6036 cases  3757 controls |
| ADHD | Psychiatric Genetics Consortium (PGC) | https://www.med.unc.edu/pgc/downloads | Cross-Disorder Group of the Psychiatric Genomics Consortium. Lancet 2013; 381: 1371-1379. PMID: 23453885 | 1947 trio cases 1947 trio pseudocontrols, 840 cases  688 controls |
| Alzheimer's disease | International Genomics of Alzheimer’s Project (IGAP) | http://www.pasteur-lille.fr/en/recherche/u744/igap/igap_download.php | Lambert et al. Nat Genet 2013; 45: 1452-1458. PMID: 24162737 | 17 008 cases  37 154 controls |
| Autism | Psychiatric Genetics Consortium (PGC) | http://www.med.unc.edu/pgc/downloads | Cross-Disorder Group of the Psychiatric Genomics Consortium. Nat Genet 2013: 45; 984-994. PMID: 23933821 | 3303 cases  3428 controls |
| Anorexia nervosa | Genetic Consortium for Anorexia Nervosa (GCAN) | http://www.med.unc.edu/pgc/downloads | Boraska, Vesna, et al. Molecular psychiatry 2014; 19(10): 1085-1094. PIMD: 24514567 | 2907 cases 14860 controls |
| Bipolar disorder | Psychiatric Genetics Consortium (PGC) | https://www.med.unc.edu/pgc/downloads | Psychiatric GWAS Consortium Bipolar Disorder Working Group. Nat Genet 2011; 43: 977-983. PMID: 21926972 | 7481 cases  9250 controls |
| Major depressive disorder | Psychiatric Genetics Consortium (PGC) | https://www.med.unc.edu/pgc/downloads | Major Depressive Disorder Working Group of the Psychiatric GWAS Consortium. Mol Psychiatr 2013; 18: 497-511. PMID: 22472876 | 9240 cases  9519 controls |
| Schizophrenia | Psychiatric Genetics Consortium (PGC) | https://www.med.unc.edu/pgc/downloads | Schizophrenia Working Group of the Psychiatric Genomics Consortium. Nature 2014; 511: 421-427. PMID: 25056061 | 36 989 cases  113 075 controls |
| Rheumatoid Arthritis |  | http://www.broadinstitute.org/ftp/pub/rheumatoid_arthritis/Stahl_etal_2010NG/ | Stahl et al Nature Genetics 2012; 44:483-489 | 5500 cases  20 000 controls |
| Forced expiratory volume in 1 second (FEV_1_) | SpiroMeta/CHARGE-Pulmonary |  | Soler Artigas et al. Nature Genetics 2011; 43: 1082-1090. PMID: 21946350 | 48 201 |
| Blood pressure: Diastolic | International Consortium of Blood Pressure (ICBP) |  | Ehret et al. (2011) Nature 478, 103-109. PMID: 21909115 | 69 395 |
| Blood pressure: Systolic | International Consortium of Blood Pressure (ICBP) |  | Ehret et al. Nature 2011; 478: 103-109. PMID: 21909115 | 69 395 |
| HbA1c | MAGIC | http://www.magicinvestigators.org/downloads/ | Soranzo et al. Diabetes 2010; 59: 3229-3239 | 46 368 |
| Coronary Artery Disease | CARDIoGRAM | http://www.cardiogramplusc4d.org/downloads/ | Schunkert et al. Nat Genet 2011; 43: 333-338. PMID: 21378990 | 22 233 cases  64 762 controls |
| HDL Cholesterol | Global Lipids Consortium | http://csg.sph.umich.edu//abecasis/public/lipids2013/ | Willer et al. Nature Genet 2013; 45: 1274-1283 | 188 577 |
| LDL Cholesterol | Global Lipids Consortium | http://csg.sph.umich.edu//abecasis/public/lipids2013/ | Willer et al. Nature Genet 2013; 45: 1274-1283 | 188 577 |
| Triglycerides | Global Lipids Consortium | http://csg.sph.umich.edu//abecasis/public/lipids2013/ | Willer et al. Nature Genet 2013; 45: 1274-1283 | 188 577 |
| Smoking status | TAG | https://www.med.unc.edu/pgc/files/resultfiles/tag.evrsmk.tbl.gz | Furberg et al Nature Genetics 2010; 42: 441-447 | 74 053 |
| Type 2 diabetes | DIAGRAM | http://diagram-consortium.org/downloads.html | Morris et al. Nat Genet 2012; 44: 981-990. PMID: 22885922 | 12 171 cases  56 862 controls |
| C-reactive protein | CHARGE Inflammation Group |  | Dehghan et al. Circulation 2011: 123: 731-738. PMID: 21300955 | 66 185 |

**Supplementary Figure 1.** Age- and sex distribution of standardized tiredness scores

**
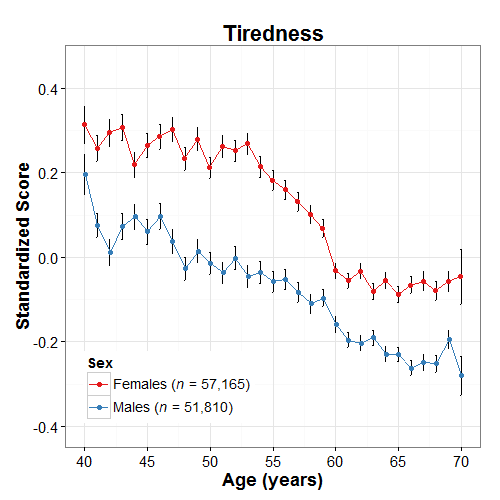
**

**Supplementary Table 2**. Frequency table for tiredness and self-rated health

|  |  | **Self-rated health** | | | |
| --- | --- | --- | --- | --- | --- |
|  |  | excellent | good | fair | poor |
| **Tiredness** | not at all | 12556 | 32258 | 6105 | 428 |
|  | several days | 4857 | 25633 | 11665 | 1921 |
|  | more than half the days | 316 | 2814 | 2531 | 695 |
|  | nearly every day | 215 | 2000 | 2710 | 1944 |

**Supplementary Figure 2a.**
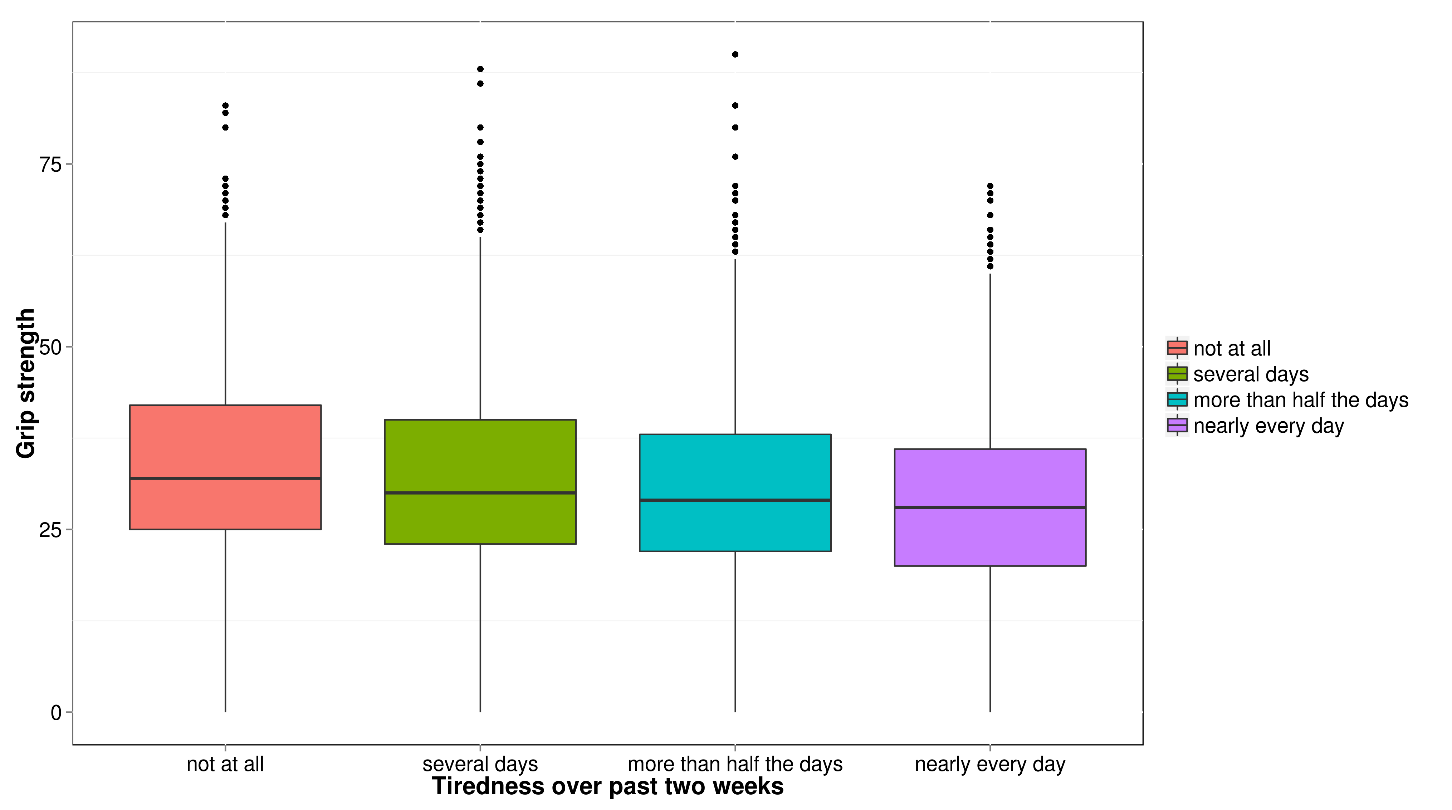
Barplot showing the distribution of grip strength for each category of tiredness.
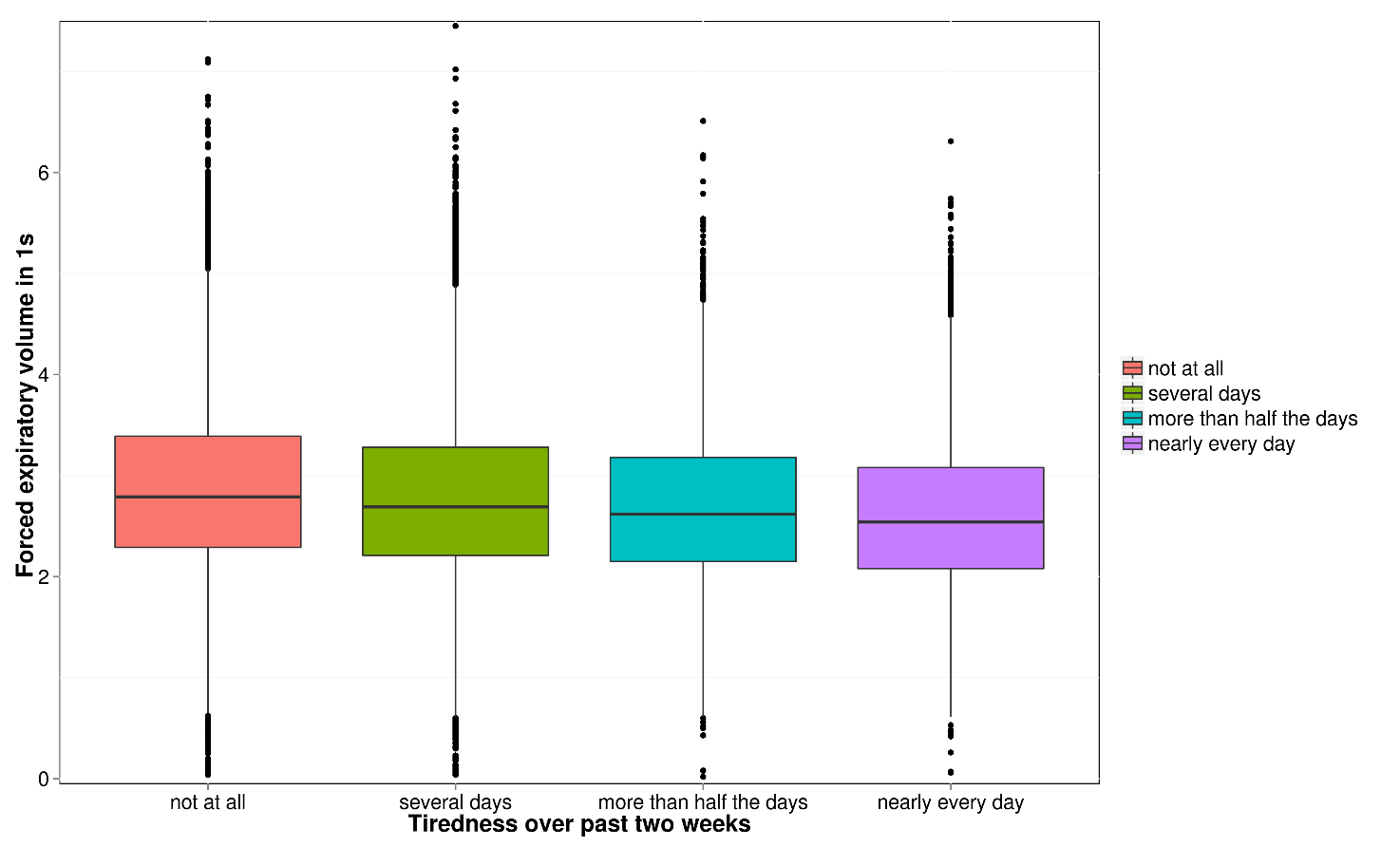


**Supplementary Figure 2b.** Barplot showing the distribution of lung function for each category of tiredness, 13 individuals with values above 7.5 litres/second have been excluded.


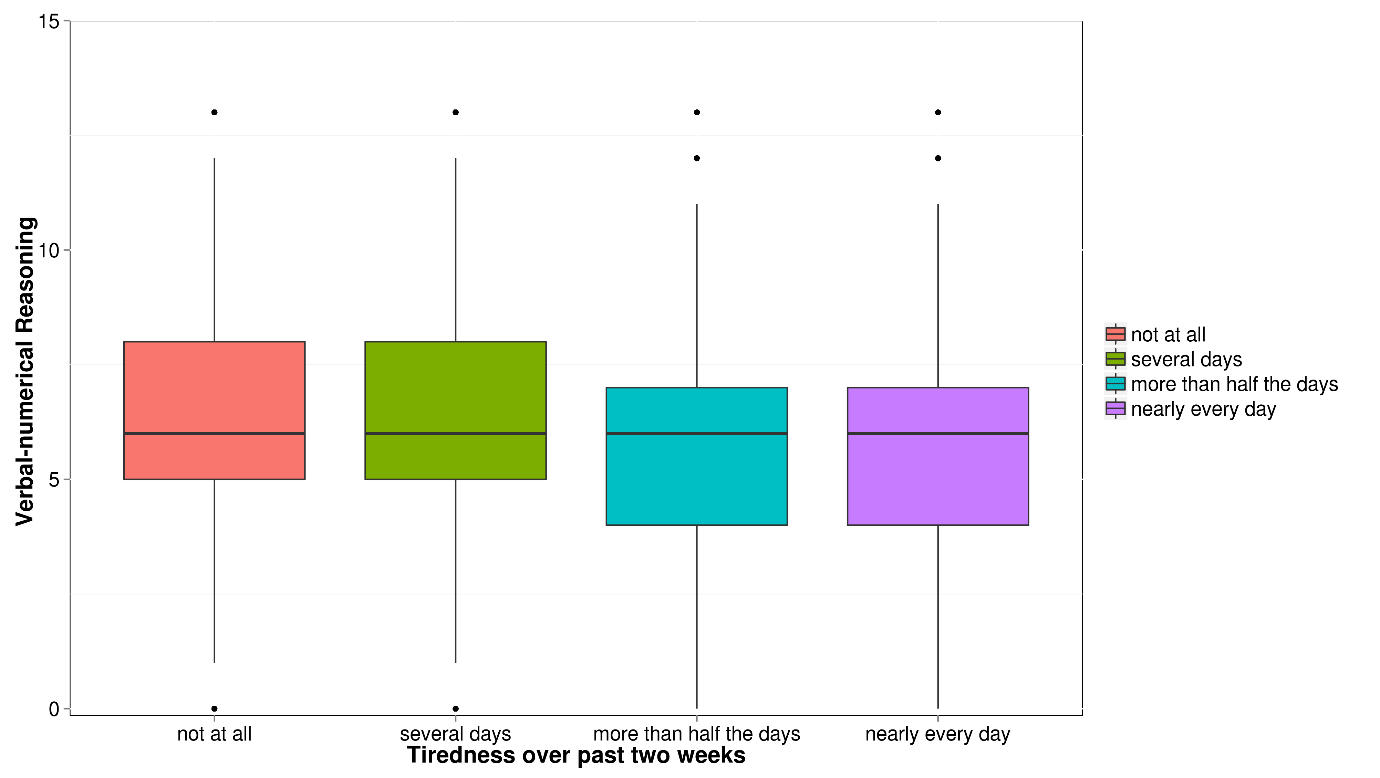


**
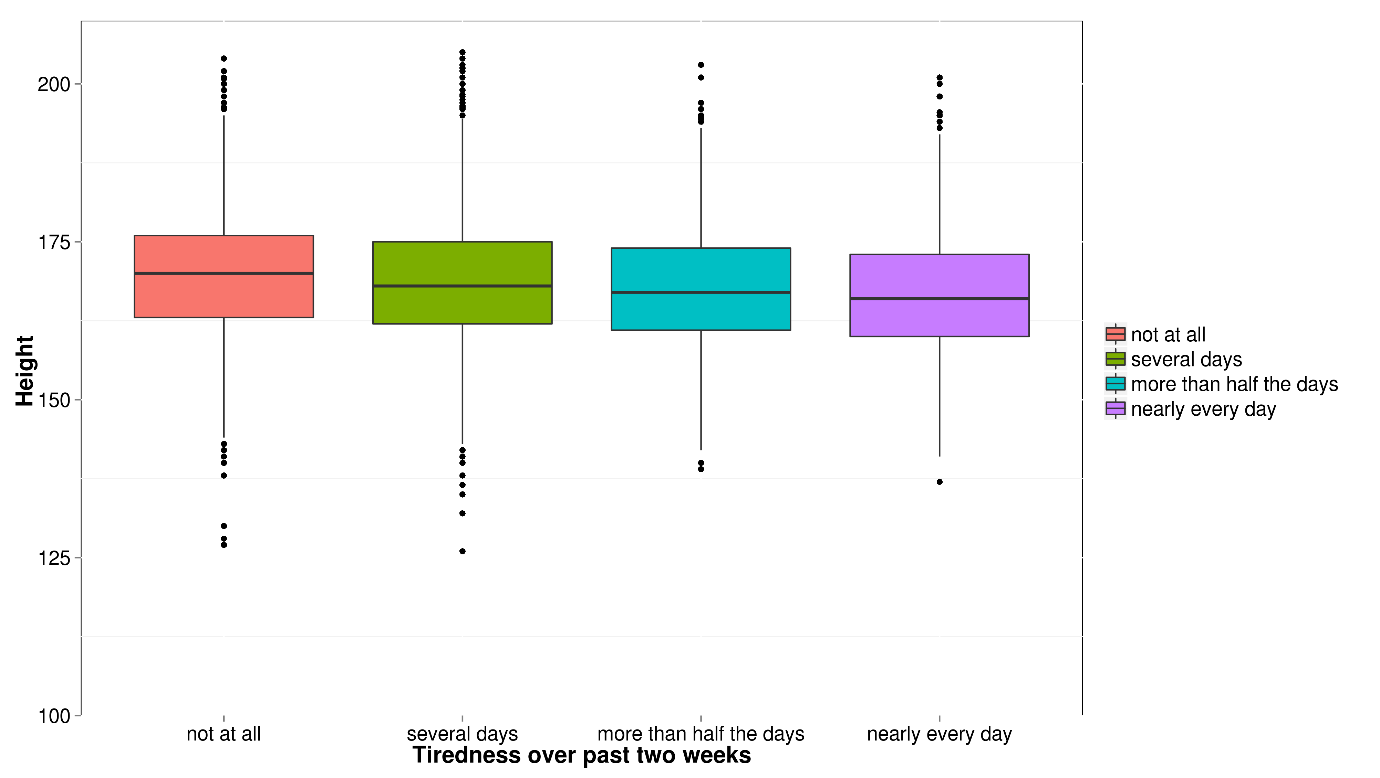
Supplementary Figure 2c.** Barplot showing the distribution of verbal-numerical reasoning for each category of tiredness.

**Supplementary Figure 2d.** Barplot showing the distribution of height for each category of tiredness.


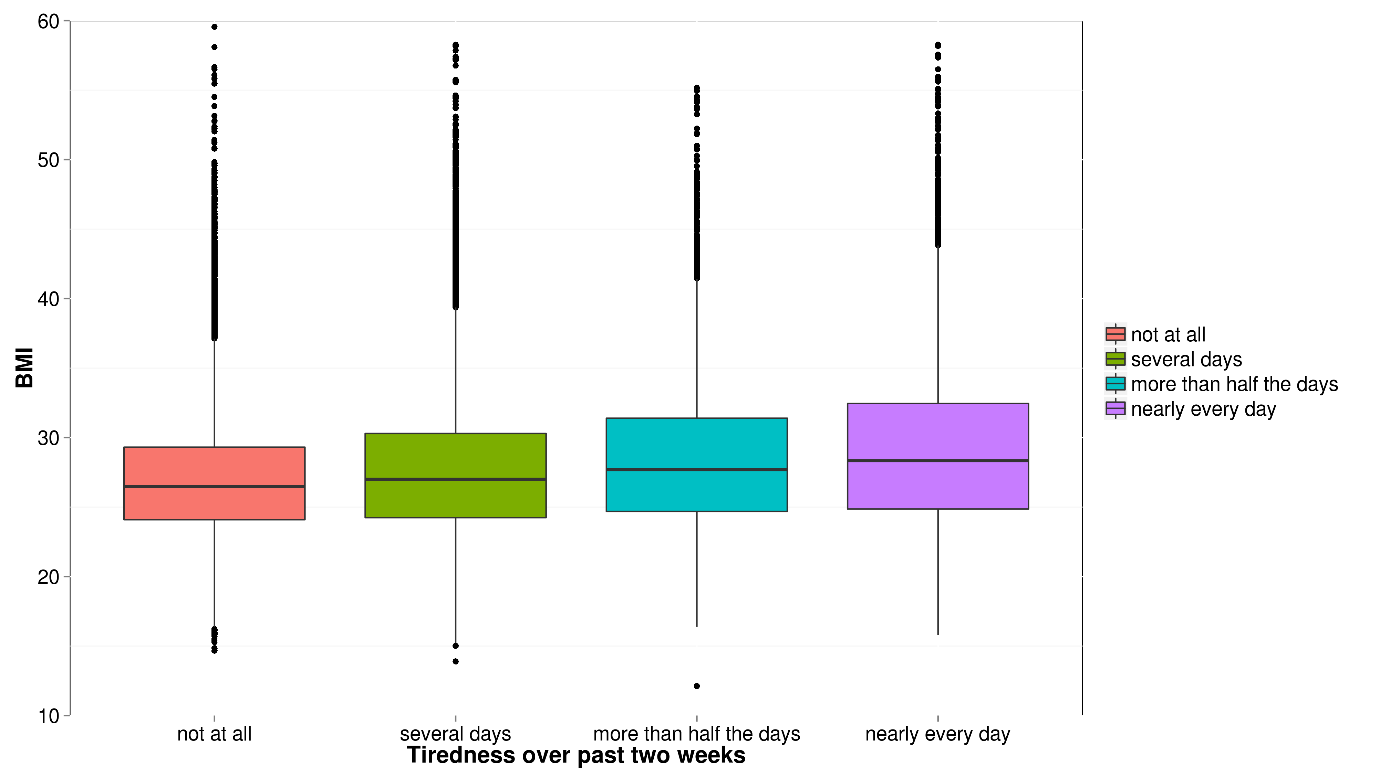


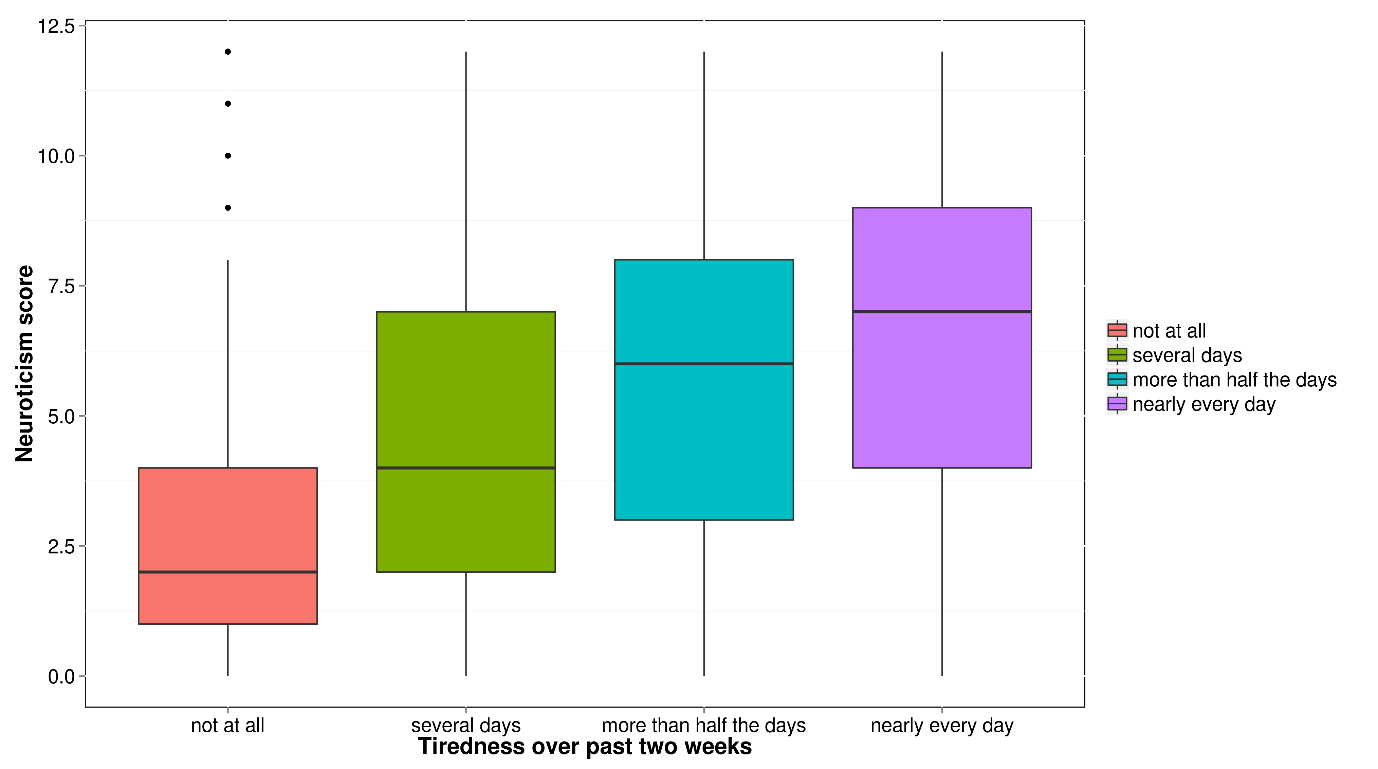
**Supplementary Figure 2e** Barplot showing the distribution of BMI for each category of tiredness.

**Supplementary Figure 2f**: Barplot showing the distribution of neuroticism for each category of tiredness.

**Supplementary Table 3**. Gene based analysis conducted on tiredness using MAGMA. Genome-wide significant gene-based hits (P<2.8 x 10-6) are shown in bold. NSNPS is the number of SNPs in the gene; Effect Size is the number of independent SNPs in the gene.

(See attached Excel spreadsheet)

**Supplementary Table 4.** Associations between polygenic profiles of health related traits, and tiredness controlling for age, sex, assessment centre, genotyping batch and array, and ten principal components for population structure. Statistically significant values (P < 0.0255) are shown in bold.

(See attached Excel spreadsheet)

**Supplementary Table 5.** Associations between polygenic profiles of health-related traits created from GWAS consortia summary data, and the UK Biobank tiredness phenotype controlling for age, sex, assessment centre, genotyping batch and array and 10 principal components for population structure. SRH models are adjusted for self-rated health; N models are adjusted for neuroticism.

(See attached Excel spreadsheet)

**Supplementary Table 6**. Associations between polygenic profiles of health-related traits created from GWAS consortia summary data, and the UK Biobank tiredness phenotype controlling for age, sex, assessment centre, genotyping batch and array and 10 principal components for population structure in a subset of individuals who have sufficient data for MDD diagnosis.

(See attached Excel spreadsheet)

**Supplementary Figure 3**

Enrichment analysis for tiredness using the 52 functional categories in the baseline model. The enrichment statistic is the proportion of heritability found in each functional group divided by the proportion of SNPs in each group (Pr(h^2^)/Pr(SNPs)). The dashed line indicates no enrichment found when Pr(h^2^)/Pr(SNPs) = 1. Statistical significance is indicated by asterisk at *P =* 0.00039.

**Supplementary Figure 4**

Enrichment analysis for tiredness using the 10 cell specific functional categories. The enrichment statistic is the proportion of heritability found in each functional group divided by the proportion of SNPs in each group (Pr(h^2^)/Pr(SNPs). The dashed line indicates no enrichment found when Pr(h^2^)/Pr(SNPs) = 1. Statistical significance is indicated by asterisk at *P =* 0.000202877.

**Supplementary Table 7.** Multivariate model predicting tiredness, including all significant polygenic profile scores together with covariates (age, sex, assessment centre, genotyping batch and array, and ten genetic principal components for population structure; covariate values not shown here). Adjusted R^2^ values refer to the polygenic profile scores only (excluding variance explained by the covariates). Statistically significant p-values (after FDR correction; threshold: P < 0.0243 are shown in bold.

|  | **All Traits** | |
| --- | --- | --- |
|  | **Adjusted R^2^ = 0.25%** | |
| **Traits** | **β** | **P** |
| BMI | 0.0164 | **0.0001** |
| Cholesterol: HDL | -0.0079 | **0.0169** |
| Cholesterol: LDL | 0.0025 | 0.4304 |
| Coronary artery disease | 0.0045 | 0.1437 |
| C-reactive protein | 0.0032 | 0.3114 |
| HbA1c | 0.0060 | 0.0537 |
| Height | -0.0015 | 0.6512 |
| Obesity | 0.0057 | 0.1773 |
| Smoking status | 0.0062 | 0.0527 |
| Triglycerides | 0.0097 | **0.0043** |
| Type 2 diabetes | 0.0051 | 0.1085 |
| Waist-hip ratio | 0.0111 | **0.0011** |
| Bipolar disorder | 0.0020 | 0.5131 |
| Childhood cognitive ability | -0.0073 | **0.0182** |
| Major depressive disorder | 0.0140 | **8.67×10^-6^** |
| Neuroticism | 0.0145 | **1.92×10^-6^** |
| Schizophrenia | 0.0254 | **2.25×10^-15^** |

**Supplementary Table 8**. Genetic correlations between allostatic load variables. Statistically significant P-values (after false discovery rate correction; threshold: P = 0.0273) are shown in bold.

(See attached Excel spreadsheet)

**Supplementary Figure 5.** A) Miami of P-values of the SNP-based association analysis of tiredness (responses to the question, “Over the past two weeks, how often have you felt tired or had little energy?”). The red line indicates the threshold for genome-wide significance (P<5 x 10-8) and the threshold for suggestive significance (P<1 x 10-5), the upper half shows the results for females and the bottom half shows the results for males. (B) Q-Q plot; pink for females and blue for males.

**
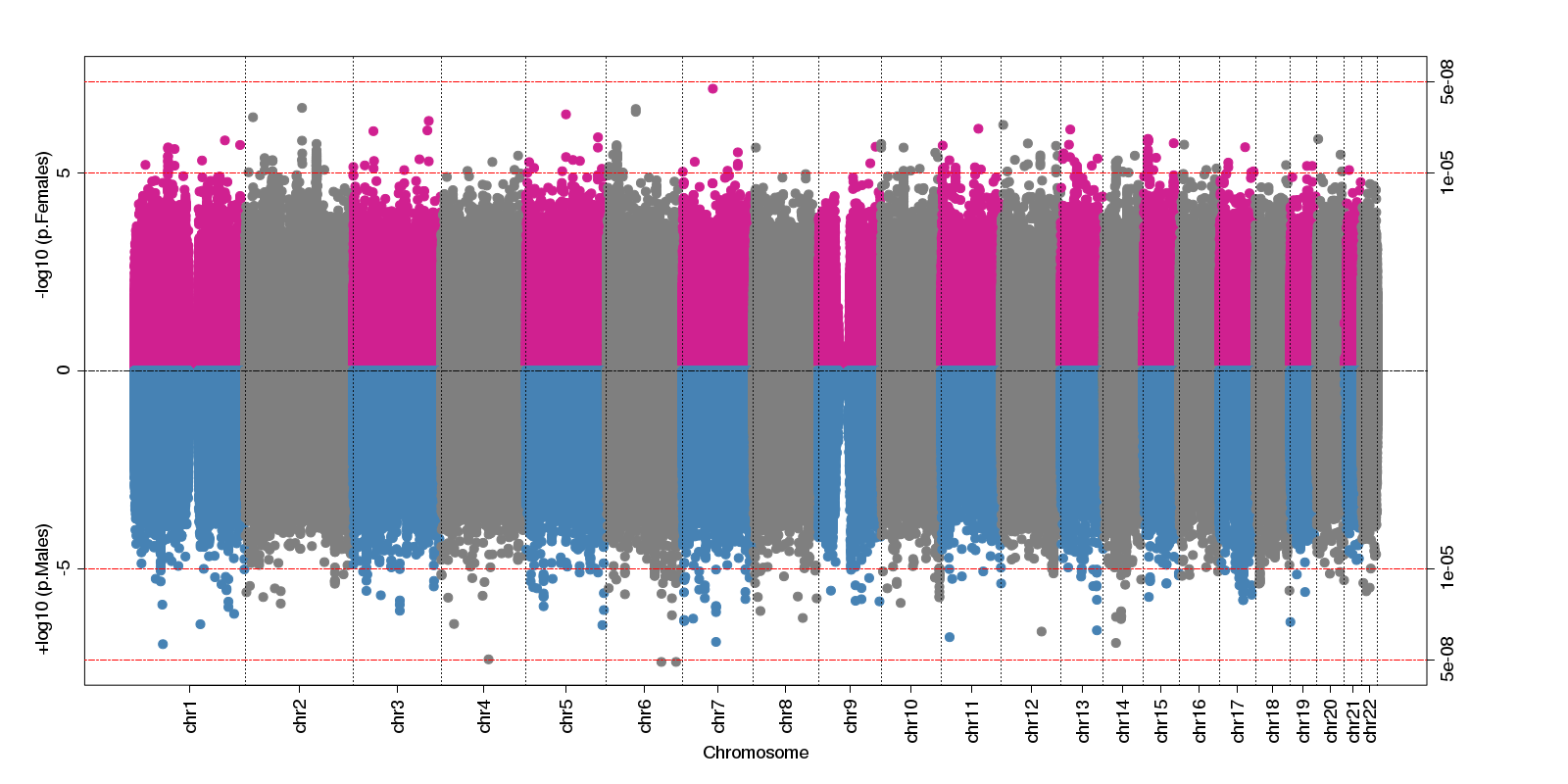

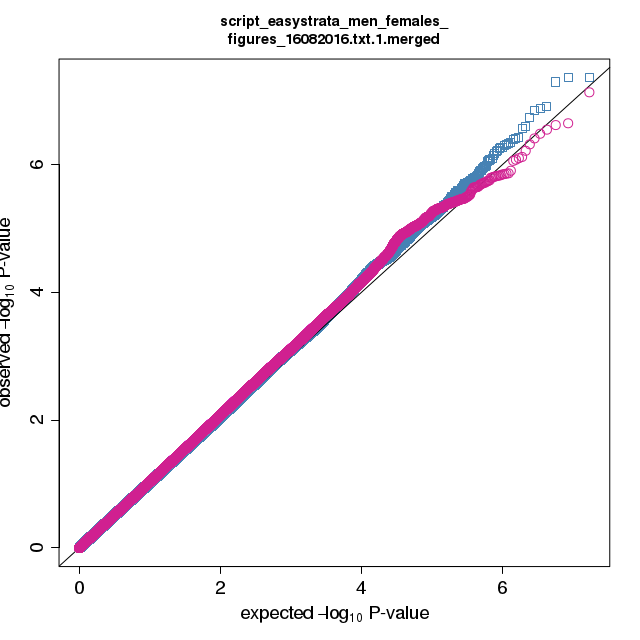
**

**Supplementary Figure 6.** Manhattan plot of the difference in effect size between males and females.

**
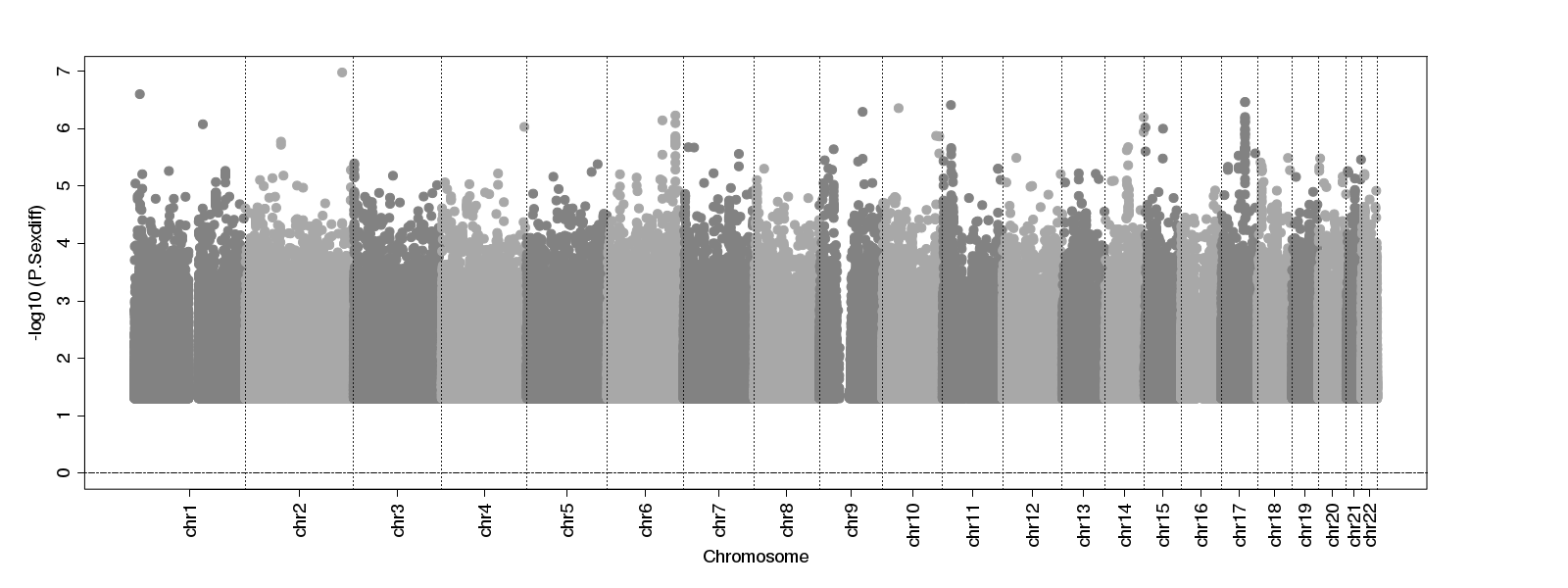
**

**
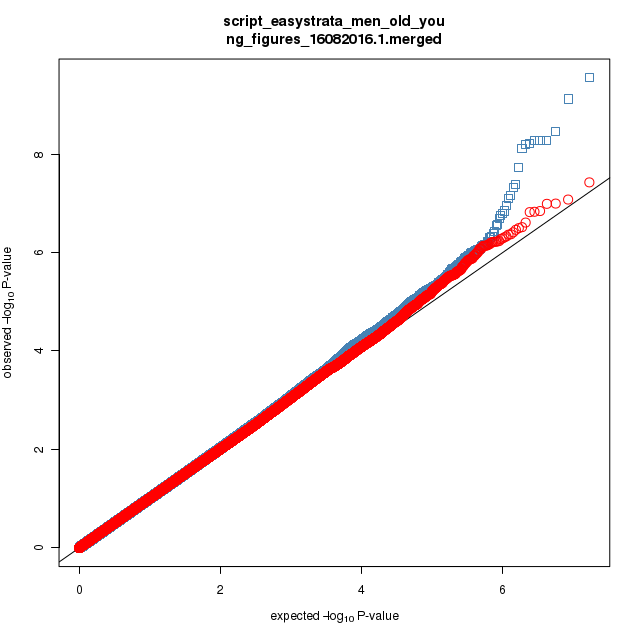

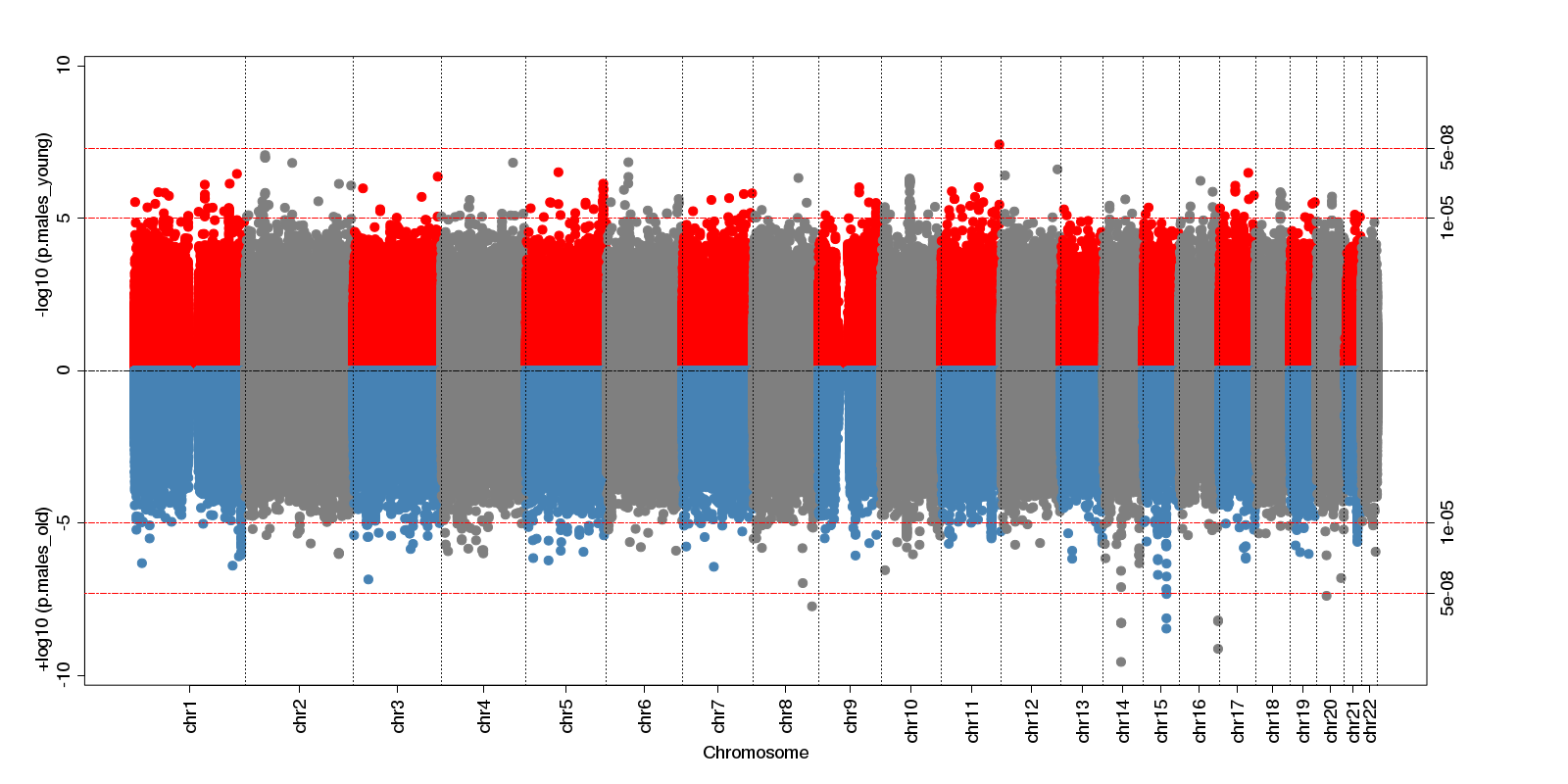
Supplementary Figure 7.** A) Miami of P-values of the SNP-based association analysis of tiredness (responses to the question, “Over the past two weeks, how often have you felt tired or had little energy?”). The red line indicates the threshold for genome-wide significance (P<5 x 10-8) and the threshold for suggestive significance (P<1 x 10-5), the upper half shows the results for females and the bottom half shows the results for males. (B) Q-Q plot; red for males aged 40-50 years and blue for males aged 60-70 years.

**
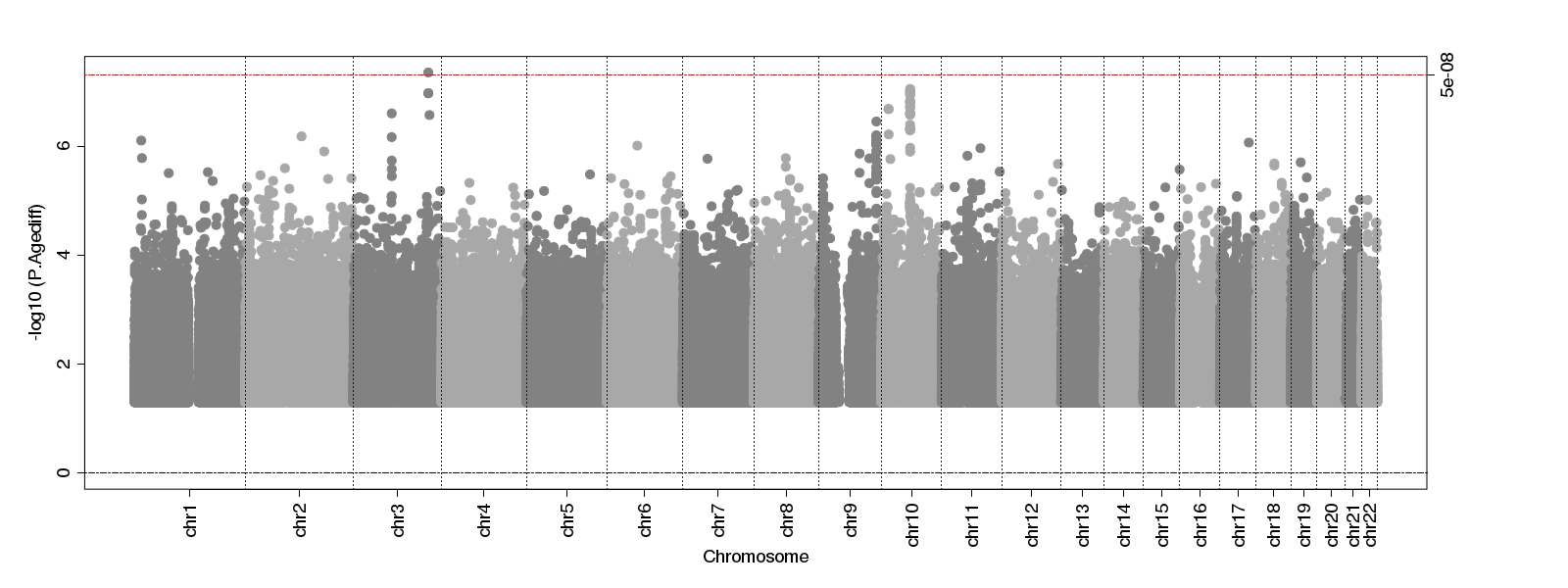
Supplementary Figure 8.** Manhattan plot of the difference in effect size between males aged 40-50 years and males aged 60-70 years.

**References**

Benjamini, Y., & Hochberg, Y. (1995). Controlling the false discovery rate: a practical and powerful approach to multiple testing. *Journal of the Royal Statistical Society Series B (Methodological), 57*(1), 289-300.

Deary, I. J., & Bedford, A. (2011). Some origins and evolution of the EPQ-R (short form) Neuroticism and Extraversion items. *Personality and Individual Differences, 50*(8), 1213-1217.

ENCODE Project Consortium. (2012). An integrated encyclopedia of DNA elements in the human genome. *Nature, 489*(7414), 57-74.

Eysenck, S. B. G., Eysenck, H. J., & Barrett, P. (1985). A revised version of the psychoticism scale. *Personality and Individual Differences, 6*(1), 21-29. doi:<http://dx.doi.org/10.1016/0191-8869(85)90026-1>

Finucane, H. K., Bulik-Sullivan, B., Gusev, A., Trynka, G., Reshef, Y., Loh, P. R., . . . Consortium, R. (2015). Partitioning heritability by functional annotation using genome-wide association summary statistics. *Nature genetics, 47*(11), 1228-1235.

Gale, C. R., Hagenaars, S. P., Davies, G., Hill, W. D., Liewald, D. C., Cullen, B., . . . Deary, I. J. (In Press). Pleiotropy between neuroticism and physical and mental health: findings from 108 038 men and women in UK Biobank. *Translational Psychiatry*.

Gusev, A., Lee, S. H., Trynka, G., Finucane, H., Vilhjálmsson, B. J., Xu, H., . . . SWE-SCZ Consortium. (2014). Partitioning heritability of regulatory and cell-type-specific variants across 11 common diseases. *The American Journal of Human Genetics, 95*(5), 535-552.

Hagenaars, S. P., Harris, S. E., Davies, G., Hill, W. D., Liewald, D. C., Ritchie, S. J., . . . Deary, I. J. (2016). Shared genetic aetiology between cognitive functions and physical and mental health in UK Biobank (N=112 151) and 24 GWAS consortia. *Mol Psychiatry*. doi:10.1038/mp.2015.225

Harris, S. E., Hagenaars, S. P., Davies, G., Hill, W. D., Liewald, D. C., Ritchie, S. J., . . . McIntosh, A. M. (2015). Molecular genetic contributions to self-rated health. *bioRxiv*, 029504.

Hnisz, D., Abraham, B. J., Lee, T. I., Lau, A., Saint-André, V., Sigova, A. A., . . . Young, R. A. (2013). Super-enhancers in the control of cell identity and disease. *Cell, 155*(4), 934-947.

Hoffman, M. M., Ernst, J., Wilder, S. P., Kundaje, A., Harris, R. S., Libbrecht, M., . . . Noble, W. S. (2012). Integrative annotation of chromatin elements from ENCODE data. *Nucleic acids research*, 1-15. doi:doi:10.1093

Kent, W. J., Sugnet, C. W., Furey, T. S., Roskin, K. M., Pringle, T. H., Zahler, A. M., & Haussler, D. (2002). The human genome browser at UCSC. *Genome research, 12*(6), 996-1006.

Lindblad-Toh, K., Garber, M., Zuk, O., Lin, M. F., Parker, B. J., Washietl, S., . . . Birney, E. (2011). A high-resolution map of human evolutionary constraint using 29 mammals. *Nature, 478*(7370), 476-482.

Schizophrenia Working Group of the Psychiatric Genomics Consortium. (2014). Biological insights from 108 schizophrenia-associated genetic loci. *Nature, 511*(7510), 421-427.

Smith, D. J., Nicholl, B. I., Cullen, B., Martin, D., Ul-Haq, Z., Evans, J., . . . Pell, J. P. (2013). Prevalence and Characteristics of Probable Major Depression and Bipolar Disorder within UK Biobank: Cross-Sectional Study of 172,751 Participants. *PLoS ONE, 8*(11), e75362. doi:10.1371/journal.pone.0075362

Trynka, G., Sandor, C., Han, B., Xu, H., Stranger, B. E., Liu, X. S., & Raychaudhuri, S. (2013). Chromatin marks identify critical cell types for fine mapping complex trait variants. *Nature genetics, 45*(2), 124-130.

Ward, L. D., & Kellis, M. (2012). Evidence of abundant purifying selection in humans for recently acquired regulatory functions. *Science, 337*(6102), 1675-1678.
