# Supplementary figures and images for: Erratum: Genetic contributions to self-reported tiredness
Source: Mol Psychiatry. 2017 Mar 21;23(3):789–90. doi: 10.1038/mp.2017.70 (PMC5822463; doi:10.1038/mp.2017.70)

## Slide 1
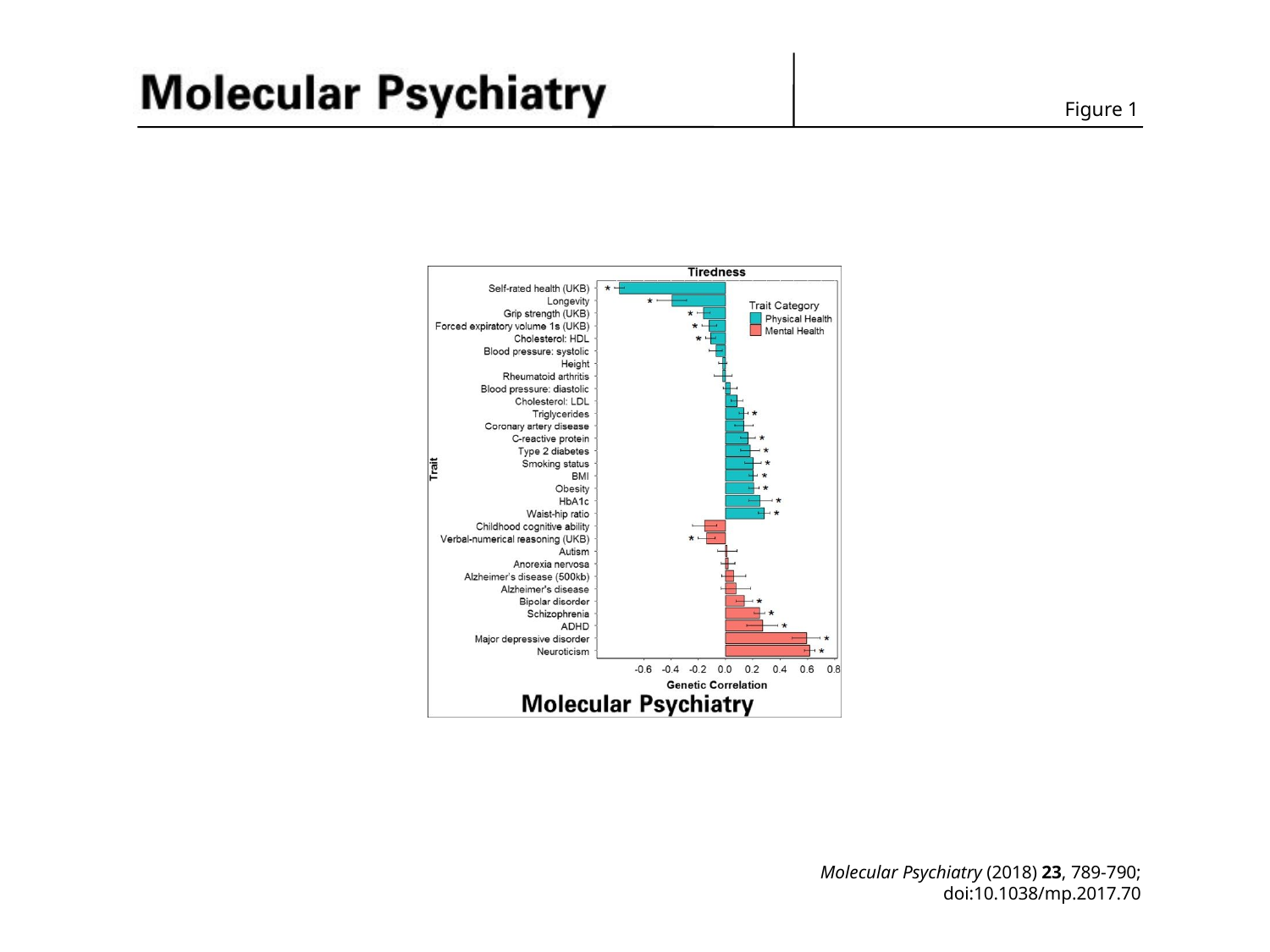

Figure 1
Molecular Psychiatry (2018) 23, 789-790;
doi:10.1038/mp.2017.70

Supplement: Supplementary file 1 — PowerPoint slide for Fig. 1 [file 41380_2018_BFmp201770_MOESM51_ESM.ppt]
